# Whole genome sequencing of a snailfish from the Yap Trench (~7,000 m) clarifies the molecular mechanisms underlying adaptation to the deep sea

**DOI:** 10.1371/journal.pgen.1009530

**Published:** 2021-05-13

**Authors:** Yinnan Mu, Chao Bian, Ruoyu Liu, Yuguang Wang, Guangming Shao, Jia Li, Ying Qiu, Tianliang He, Wanru Li, Jingqun Ao, Qiong Shi, Xinhua Chen

**Affiliations:** 1 Key Laboratory of Marine Biotechnology of Fujian Province, Institute of Oceanology, Fujian Agriculture and Forestry University, Fuzhou, Fujian, China; 2 Shenzhen Key Lab of Marine Genomics, Guangdong Provincial Key Lab of Molecular Breeding in Economic Animals, BGI Academy of Marine Sciences, BGI Marine, Shenzhen, Guangdong, China; 3 Key Laboratory of Marine Biogenetic Resources, Third Institute of Oceanography, Ministry of Natural Resources, Xiamen, Fujian, China; 4 Southern Marine Science and Engineering Guangdong Laboratory (Zhuhai), Zhuhai, Guangdong, China; The University of North Carolina at Chapel Hill, UNITED STATES

## Abstract

Hadal environments (depths below 6,000 m) are characterized by extremely high hydrostatic pressures, low temperatures, a scarce food supply, and little light. The evolutionary adaptations that allow vertebrates to survive in this extreme environment are poorly understood. Here, we constructed a high-quality reference genome for Yap hadal snailfish (YHS), which was captured at a depth of ~7,000 m in the Yap Trench. The final YHS genome assembly was 731.75 Mb, with a contig N50 of 0.75 Mb and a scaffold N50 of 1.26 Mb. We predicted 24,329 protein-coding genes in the YHS genome, and 24,265 of these genes were successfully functionally annotated. Phylogenetic analyses suggested that YHS diverged from a Mariana Trench snailfish approximately 0.92 million years ago. Many genes associated with DNA repair show evidence of positive selection and have expanded copy numbers in the YHS genome, possibly helping to maintain the integrity of DNA under increased hydrostatic pressure. The levels of trimethylamine N-oxide (TMAO), a potent protein stabilizer, are much higher in the muscles of YHS than in those of shallow-water fish. This difference is perhaps due to the five copies of the TMAO-generating enzyme flavin-containing monooxygenase-3 gene (*fmo3*) in the YHS genome and the abundance of trimethylamine (TMA)-generating bacteria in the YHS gut. Thus, the high TMAO content might help YHS adapt to high hydrostatic pressure by improving protein stability. Additionally, the evolutionary features of the YHS genes encoding sensory-related proteins are consistent with the scarce food supply and darkness in the hadal environments. These results clarify the molecular mechanisms underlying the adaptation of hadal organisms to the deep-sea environment and provide valuable genomic resources for in-depth investigations of hadal biology.

## Author summary

Hadal environments (depths below 6,000 m) are characterized by extremely high hydrostatic pressures, low temperatures, a scarce food supply, and little light. Fish are the only vertebrates inhabiting the hadal zone, and hadal snailfishes have been found in at least five geographically separated marine trenches. However, the genetic mechanisms that allow vertebrates to live in such extreme conditions are not well understood. Here, we constructed a high-quality reference genome for Yap hadal snailfish (YHS) captured at a depth of ~7,000 m in the Yap Trench, using long reads obtained by Pacific Biosciences Sequel sequencing. Comparative genomic analyses revealed that many genes associated with DNA repair show evidence of positive selection and have expanded copy numbers in the YHS genome, which potentially reflect the difficulty of maintaining DNA integrity under high hydrostatic pressure. Moreover, the five copies of the trimethylamine N-oxide (TMAO)-generating enzyme flavin-containing monooxygenase-3 gene (*fmo3*) and the abundance of trimethylamine (TMA)-generating bacteria in the YHS gut could provide enough TMAO to improve protein stability under hadal conditions. In addition, characteristics of the YHS sensory system genes were consistent with the scarce food supply and darkness in the hadal zone. Our results provide new insights into the molecular mechanisms underlying the adaptation of hadal organisms to the deep-sea environment and valuable genomic resources that will help further clarify hadal adaptations.

## Introduction

The hadal zone (6,000–11,000 m deep) is composed mainly of deep trenches, and is usually characterized by extremely high hydrostatic pressures, low temperatures, a scarce food supply, a lack of light, and geographical isolation [1,2]. Hydrostatic pressure is the most conspicuous environmental gradient in the deep sea, increasing by approximately 0.1 megapascals (MPa) with every ten meters of depth, and reaching ∼100 MPa in the deepest hadal zone [3]. The temperatures in the hadal zone range from 1 to 2.5°C, and the light intensity there is too low to sustain photosynthetic production [4]. The food resources in the hadal zone are mainly supplemented by surface-derived carrion falls, which implies that the food in the hadal zone is much more limited than that in shallower regions [1,5]. These harsh living conditions form a unique deep ocean trench ecosystem with an endemic faunal community distinct from those in surrounding deep-sea environments [6].

Environmental extremes in the hadal zone influence the physiological and biochemical processes of marine organisms [7]. Comparative studies have revealed that the pressure sensitivities of some structural proteins, membrane-based systems, and enzymes differ markedly between deep*-* and shallow-living species [2,3,8]. For example, the volume associated with α-actin polymerization in two deep-sea fish species (*Coryphaenoides yaquinae* and *C*. *armatus*) is markedly smaller than that in congeneric shallow-living fishes (*C*. *cinereus* and *C*. *acrolepis*) [2]. Amino acid substitutions also occur during adaptation to hydrostatic pressures, and these substitutions affect protein-protein interactions or ligand binding [9]. Substitutions in lactate dehydrogenase in deep-sea fishes help the enzyme better tolerate high hydrostatic pressures [7]. Another potential mechanism of pressure adaptation involves several small organic solutes, which are referred to as “pyrolites” [10,11]. Pyrolites enhance the structural stability and binding ability of proteins by altering water molecule structure to reduce its tendency to pressurize [12]. Trimethylamine N-oxide (TMAO), a potent protein stabilizer commonly found in the muscles of marine fish species, can counteract the effects of hydrostatic pressure on enzyme kinetics and protein stability [10]. In deep-sea teleosts, a striking correlation exists between capture depth and TMAO content [13,14]. Moreover, at low temperatures, the DNA and RNA strand structures tend to tighten, which hinders interaction with enzymes involved in DNA replication, transcription, and translation and thus disrupts the transcription and translation processes [9].

The Liparidae (the snailfish; Scorpaeniformes), which includes more than 400 species, is probably the most bathymetrically and geographically widespread family of marine fish [15]. Liparidae has the widest depth range of all marine fish species, with habitats ranging from intertidal to depths greater than 8,000 m [16]. Hadal snailfishes have been found in at least five geographically separated marine trenches. The deepest snailfish recorded to date is *Abyssobrotula galatheae*, which was retrieved from the Puerto Rico Trench at a depth of 8,370 m in 1970 [17]. Other hadal snailfishes have been found in the Kermadec, Mariana, Kuril-Kamchatka, and Japan trenches at depths of ~6,660–7,966 m [15,18]. Snailfishes from various hadal trenches possess many similar characteristics, such as transparent skin and peritoneum, a pinkish-white body, internal organs that are visible through the skin and thin abdominal walls [15]. However, the molecular mechanisms that allow snailfishes to survive in extreme hadal environments are poorly understood, mainly due to the limited availability of genetic data. To date, only the genome assembly of the Mariana hadal snailfish (*Pseudoliparis swirei*) captured at a depth of 7,034 m in the Mariana Trench has been reported, exploring the adaptive characteristics related to the hadal environment [19].

In this study, we sequenced and assembled a reference genome for a species of hadal snailfish, collected at a depth of 6,903 m in the Yap Trench (tentatively named Yap hadal snailfish; YHS) using advanced single-molecule real-time (SMRT) sequencing. Comparative genomic analyses among YHS, Mariana hadal snailfish, and other sequenced shallow-water fish species were performed to identify genetic changes associated with adaptations to the hadal environment. The gut microbiota of YHS was also analyzed to explore its correlation with deep-sea adaptation. Our results provide new insights into the molecular mechanisms underlying adaptation of hadal organisms to the deep-sea environment and valuable genomic resources for further in-depth investigations of hadal biology.

## Results

### Characterization of the hadal snailfish from the Yap Trench and its genome sequencing

**Fig 1:**
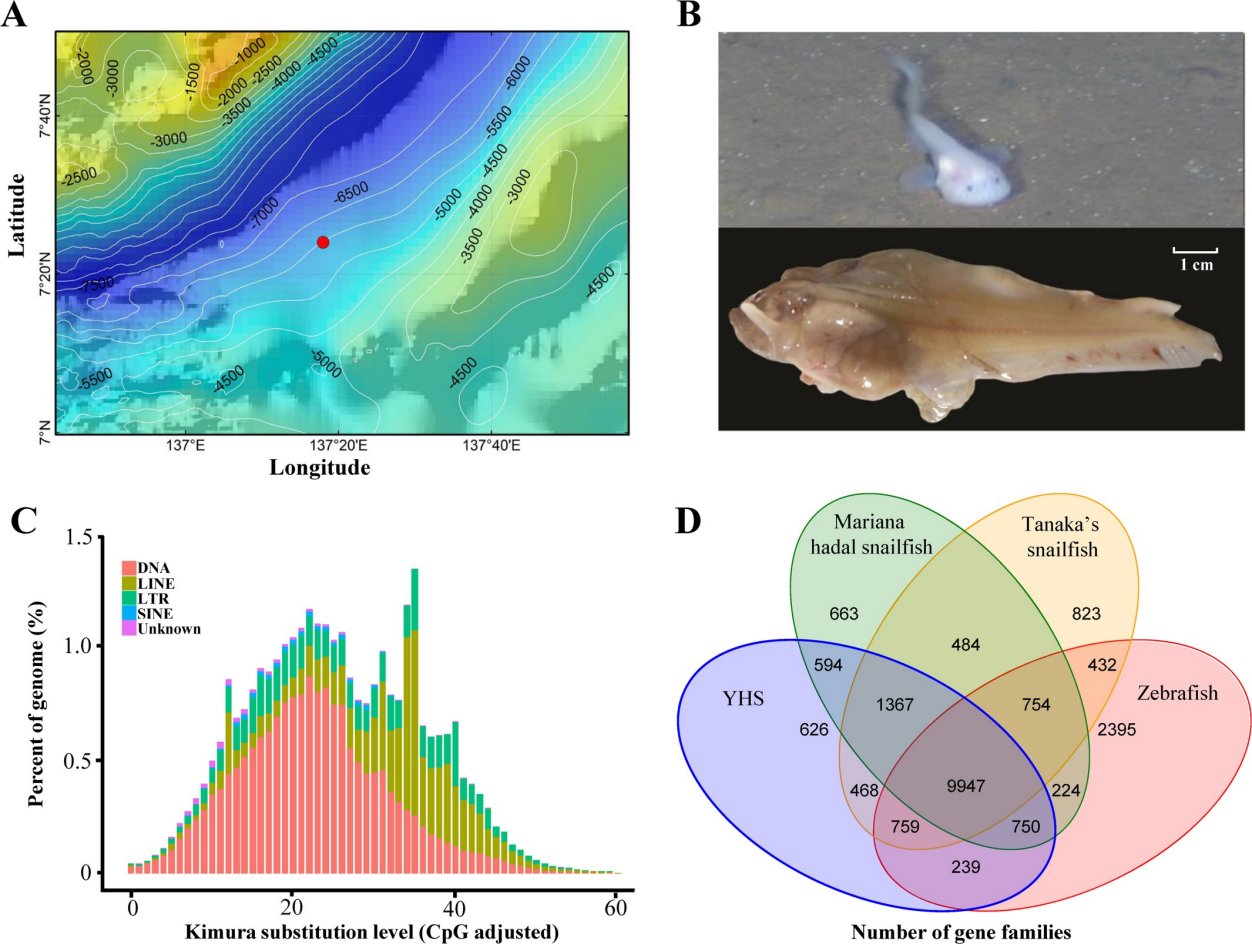
Collection and morphology of Yap hadal snailfish and its genomic characteristics. (A) Bathymetric map of the Yap Trench. The red dot indicates the location at which the two hadal snailfishes were caught by the Chinese manned submersible Jiaolong. The bathymetric map was obtained from GEBCO Compilation Group (2020) GEBCO 2020 Grid (doi:10.5285/a29c5465-b138-234d-e053-6c86abc040b9). (B) Yap hadal snailfish (YHS) in situ at 6,903 m (above) and after capture (below). (C) Distribution of TE families across the YHS genome: DNA transposons (DNA), long interspersed nuclear elements (LINEs), long terminal repeats (LTRs), short interspersed nuclear elements (SINEs), and unknown TEs (unknown). (D) Venn diagram showing shared and unique gene families across YHS, Mariana hadal snailfish, Tanaka’s snailfish, and zebrafish.

Two hadal snailfishes were collected at a depth of 6,903 m in the Yap Trench (Western Pacific Ocean; 137.3°E, 7.4°N; Fig 1A) during an expedition of the Chinese manned submersible Jiaolong (National Deep Sea Center, China). Both individuals had enlarged stomachs and livers, and these internal organs were visible through the skin and peritonea (Fig 1B). Their eyes are markedly smaller than the orbit and almost enter the dorsal profile of the head. The two individuals share many morphological characteristics with other hadal snailfishes, including pinkish-white bodies, transparent skins and peritonea, and absent pseudobranchia [15,18,19]. Phylogenetic analyses based on 16S rRNA and cytochrome c oxidase subunit I (COI) genes indicated that the snailfish from the Yap Trench fall into the same clade as the hadal snailfish from the Mariana Trench (S1 Fig). Both morphological observations and phylogenetic analyses showed that our specimens are highly similar to the Mariana hadal snailfish [15,19], and tentatively named Yap hadal snailfish (YHS).

We sequenced the genome of a YHS specimen and obtained 81.82 gigabases (Gb) of PacBio reads and 44.83 Gb of Illumina raw reads, which correspond to 99.34- and 59.87-fold coverage of the entire genome (S1 Table), respectively. The estimated size of the YHS genome is approximately 815.59 megabases (Mb), and the genome exhibits 0.61% heterozygosity (S2 Table). A total 129.70 Gb of PacBio subreads (80.87 Gb) and Illumina clean reads (44.08 Gb) were used for the *de novo* assembly of the YHS genome. The final genome assembly is approximately 731.75 Mb, which includes 1,271 scaffolds with a contig N50 of 0.75 Mb and a scaffold N50 of 1.26 Mb (Tables 1 and S3). Importantly, about 95.70% of the high-quality clean reads map to the genome assembly, which accounts for 96.17% of the complete assembly (S4 Table). In addition, 228 of the 248 highly conserved core proteins identified with the Core Eukaryotic Genes Mapping Approach (CEGMA; 91.94%), as well as 90.30% of all complete Actinopterygii Benchmarking Universal Single-Copy Orthologs (BUSCOs), were identified in our assembly (S5 and S6 Tables).

**Table 1:** Statistics of the genome assembly and annotation of Yap hadal snailfish.

Genome assembly	Data
Contig N50 size (Mb)	0.75
Scaffold N50 size (Mb)	1.26
Estimated genome size (Mb)	815.59
Assembled genome size (Mb)	731.75
Genome coverage (×)	159.21
Longest scaffold (bp)	8,357,545
**Genome annotation**	
Number of protein-coding genes	24,329
Number of annotated functional genes	24,265
Repeat content (%)	53.61

### Characterization of the Yap hadal snailfish genome

The GC content of the YHS genome assembly is 43.92% (S7 Table), the heterozygous single nucleotide polymorphism (SNP) rate is 0.2078%, and the homologous SNP rate is 0.0022% (S8 Table). The repetitive elements comprise 53.61% of the genome assembly (S9 Table), which is a much higher percentage of the genome than those found for the other sequenced snailfishes, including Mariana hadal snailfish (36.38%) and Tanaka’s snailfish (24.09%) [19]. The repetitive elements are comprised mostly of transposable elements (TEs), which account for 48.47% of the YHS genome (Fig 1C and S10 Table). Most of the TEs are long interspersed elements (LINEs; 23.98% of the genome), DNA transposons (13.54%), long terminal repeats (LTRs; 13.03%), or short interspersed elements (SINEs; 0.40%). We identified 2.14 Mb of noncoding RNAs (ncRNAs) in the YHS genome, including microRNAs (miRNAs), ribosomal RNAs (rRNAs), small nuclear RNAs (snRNAs), and transfer RNAs (tRNAs), which account for 0.29% of the genome assembly (S11 Table).

After characterizing the repetitive sequences and ncRNAs, we predicted 24,329 protein-coding genes in the final assembled genome (S12 Table), and 18,537 of these genes (76.19%) are supported by transcriptome data (S2 Fig and S13 Table). The number of protein-coding genes identified in the YHS genome is similar to previously published numbers reported from other diploid teleost genomes (S14 Table), including Mariana hadal snailfish (25,262 genes), Tanaka’s snailfish (*Liparis tanakae*; 23,776 genes), large yellow croaker *(Larimichthys crocea*; 22,274 genes), and zebrafish (*Danio rerio*; 25,619 genes). On average, the transcript length, coding sequence length, and intron length of the protein-coding genes in the YHS genome are 10,675 bp, 1,420 bp, and 1,249 bp, respectively. Moreover, each gene contains an average of 8.41 exons, which is similar to reports from other teleosts (S3 Fig). Finally, we successfully annotated 24,265 of the predicted protein-coding genes (99.74%; S15 Table).

### Gene family and genome evolution

**Fig 2:**
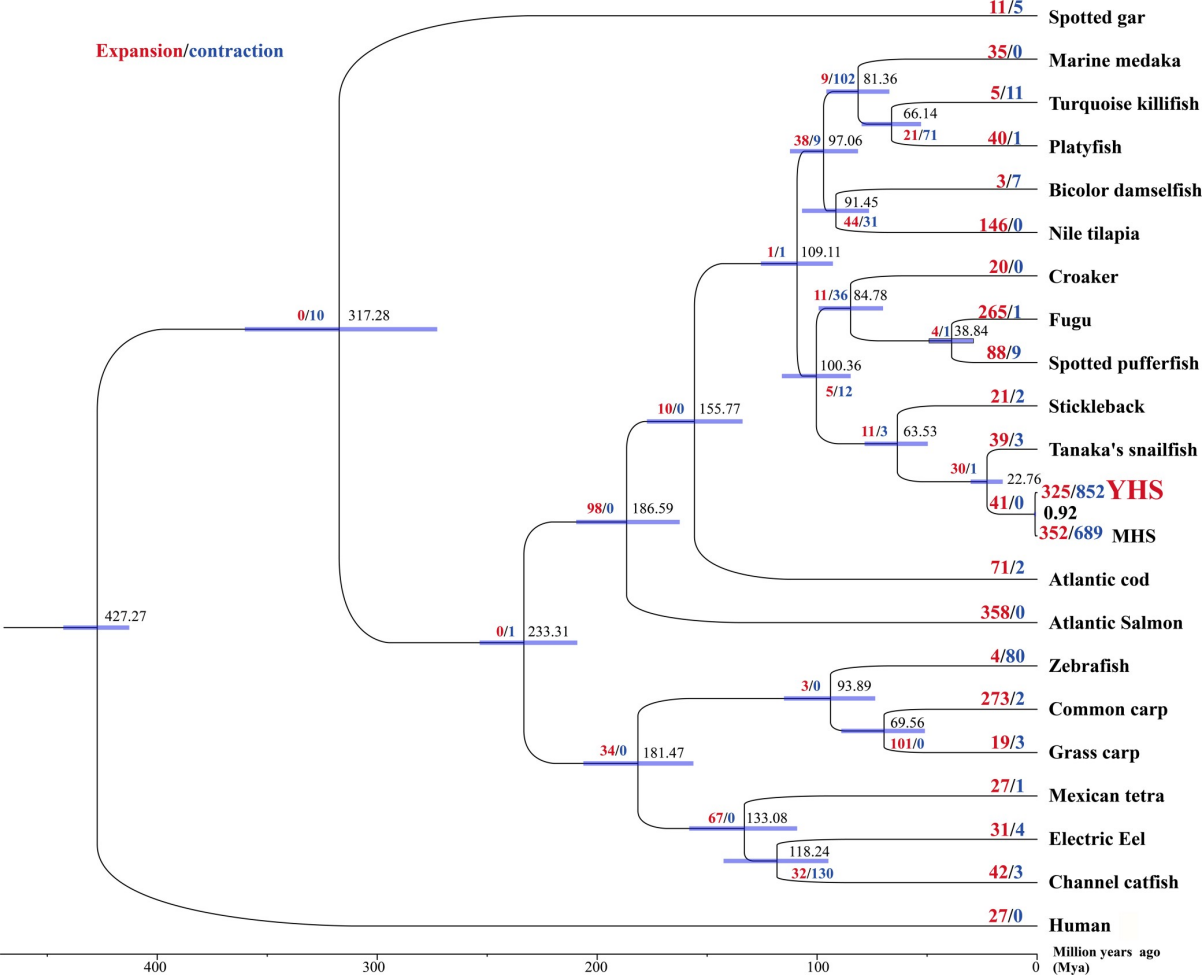
Coalescent species tree and divergence time estimation for Yap hadal snailfish and 20 other teleost species. Human served as the outgroup species. The purple rectangle bar at each node indicates the 95% confidence interval. The numbers at nodes represent estimated divergence times (Mya), and the numbers on branches indicate the event of gene family expansion (red) and contraction (blue). YHS: Yap hadal snailfish, MHS: Mariana hadal snailfish.

Across 22 representative vertebrate genomes (21 teleosts and human, S16 Table and S4 Fig), we identified 25,787 gene families and 259 shared single-copy gene families (S5 Fig). A total of 144 gene families were found only in the YHS genome. We found a high degree of gene-family overlap among YHS, Mariana hadal snailfish, Tanaka’s snailfish, and zebrafish with a core set of 9,947 gene families (Fig 1D). A coalescent species tree based on 45 partitioned datasets of the nonrecombinant single-copy orthologous genes and a dataset of concatenated single-copy orthologous genes (10,697 amino acids), recovered YHS and Mariana hadal snailfish as sister to a clade comprising Tanaka’s snailfish (Fig 2). The estimated time of divergence between our YHS and Mariana hadal snailfish was approximately 0.92 million years ago (Mya), whereas that between the two hadal snailfishes and Tanaka’s snailfish was approximately 22.76 Mya (Fig 2), which is consistent with a previous report [19]. Furthermore, 852 gene families are significantly contracted and 325 are significantly expanded in the YHS genome (Fig 2).

Compared with four shallow-water teleosts, 1,621 positively selected genes (PSGs) were identified in the YHS genome. Gene Ontology (GO) enrichment analysis revealed that 19 GO terms are overrepresented among these PSGs (level 4; S17 Table), and these terms primarily include cellular nitrogen compound metabolic process (356 genes), cellular aromatic compound metabolic process (348 genes), organic cyclic compound metabolic process (352 genes), heterocycle metabolic process (349 genes), nucleic acid binding (304 genes), and DNA repair (34 genes). Only one Kyoto Encyclopedia of Genes and Genomes (KEGG) pathway, “cytokine-cytokine receptor interaction,” is significantly enriched with six PSGs (*bmp2*, *csf3r*, *cxcr3*, *il11ra*, *il12rb2*, and *ngfr*).

### DNA repair capacity

**Fig 3:**
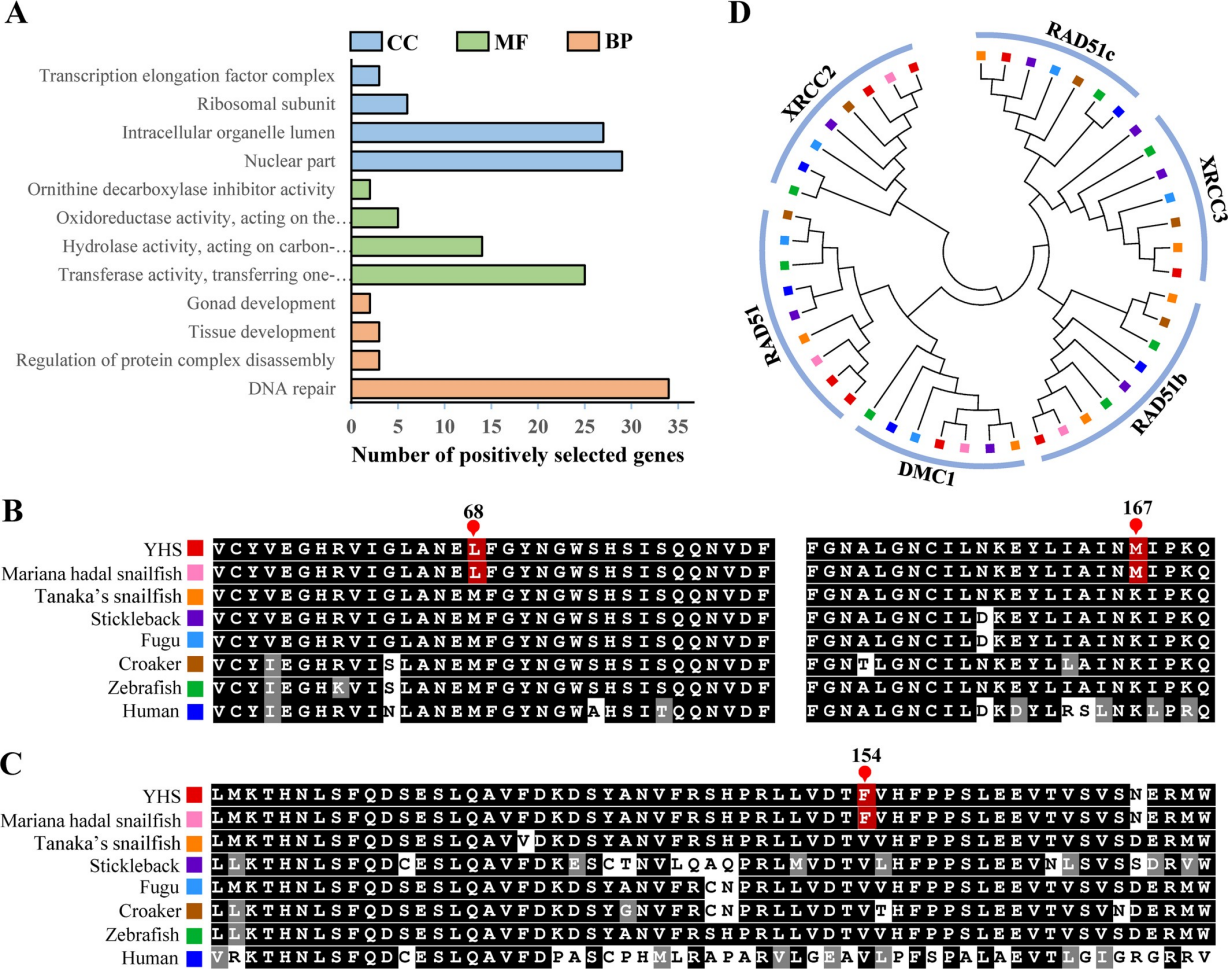
Genomic indications of enhanced DNA repair mechanisms in the Yap hadal snailfish (YHS). (A) Gene ontology enrichment of the positively selected genes from YHS (level 4). CC, cellular component, MF, molecular function, BP, biological process. (B, C) Partial alignment of the (B) RAD52 and (C) RAD9A amino acid sequences from various representative teleosts and human. Amino acids unique to two hadal snailfishes (YHS and Mariana hadal snailfish) are highlighted in red; positions within each sequence are given above. (D) A maximum-likelihood tree showing RAD51 and RAD51 paralog genes (RAD51b, RAD51c, XRCC2, XRCC3, and DMC1). The leaf-node colors correspond to the species given in panels (B) and (C).

High hydrostatic pressure can cause DNA breakage and damage [9,20]. Consistent with this, the GO term “DNA repair” is enriched with 34 PSGs (Fig 3A), including *rad52*, *rad9a*, *ercc1*, *exo1*, *pms1*, and *polk* (S18 Table). Specifically, both YHS and Mariana hadal snailfish have the same two high-confidence amino acid substitutions in the DNA repair protein RAD52 homolog (RAD52) compared with the corresponding completely conserved amino acids in Tanaka’s snailfish, large yellow croaker, zebrafish, stickleback (*Gasterosteus aculeatus*), and human (*Homo sapiens*): a methionine-to-leucine substitution at position 68 and a lysine-to-methionine substitution at position 167 (Fig 3B). RAD9A, a DNA damage checkpoint protein, is also different in the genomes of YHS and Mariana hadal snailfish compared with those of other examined vertebrates (a valine-to-phenylalanine substitution at position 154; Fig 3C). Moreover, a Pfam domain analysis revealed that the YHS genome includes eight RAD51 paralog genes (S19 Table) and more copies of *rad51* and *xrcc2* than other teleost genomes (Fig 3D).

### Protein stabilization

**Fig 4:**
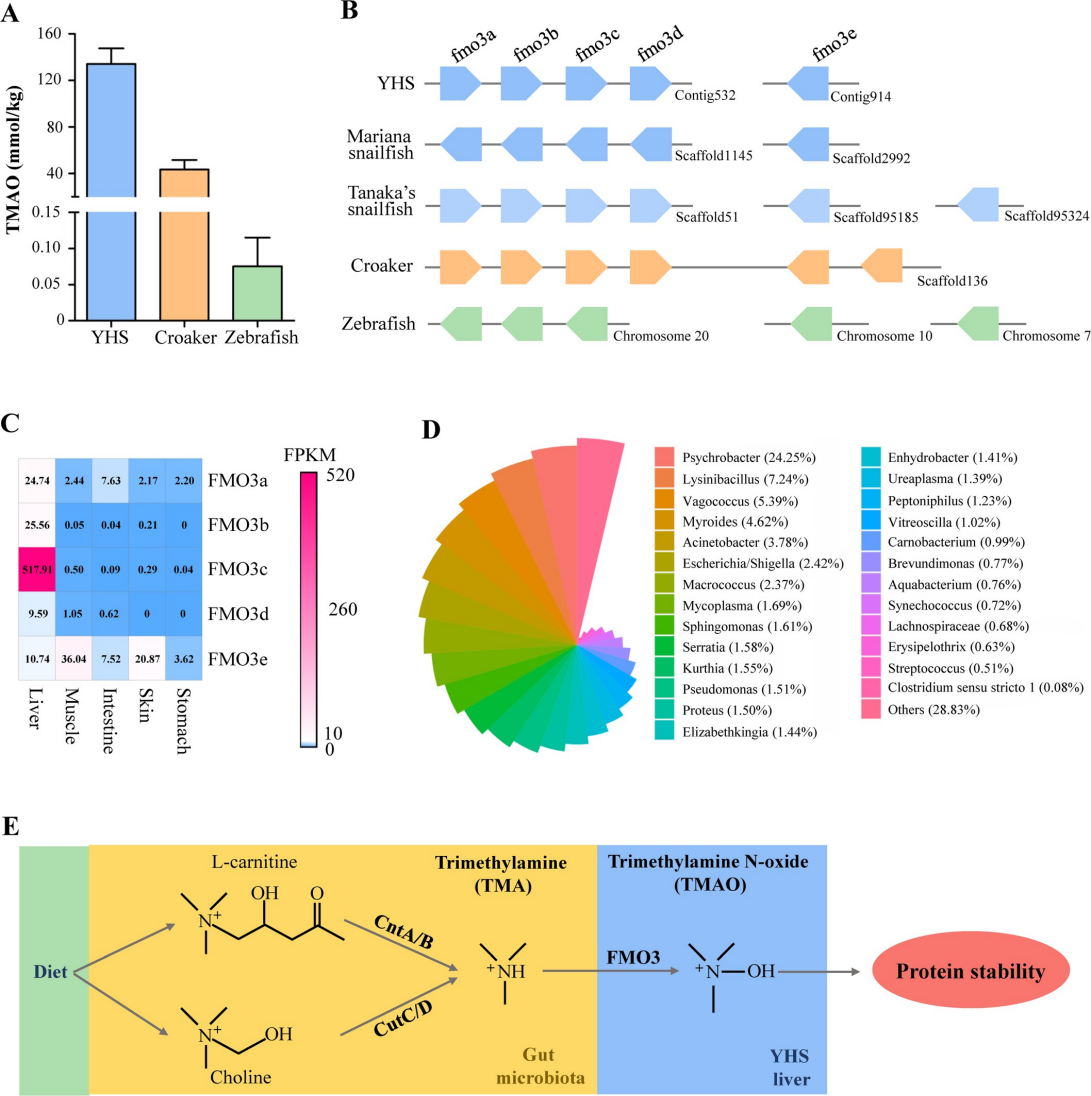
Potential mechanism of TMAO-mediated protein stabilization in Yap hadal snailfish. (A) Muscle TMAO contents (mmol/kg wet mass) in three teleosts. (B) Arrangement of the TMAO-generating enzyme *fmo3* genes in the genomes of Yap hadal snailfish (YHS), Mariana hadal snailfish, Tanaka’s snailfish, large yellow croaker, and zebrafish. (C) Relative expression of *fmo3* genes in the liver, muscle, intestine, skin, and stomach of YHS. Gene expression was quantified as fragments per kilobase of transcript per million fragments mapped (FPKM) values. (D) Bacterial communities in the YHS gut identified using the Silva database. (E) Proposed TMAO biosynthesis pathway in the YHS. Through this pathway, both the gut microbiota and five copies of *fmo3* help to maintain a high TMAO levels and thus improve protein stability to ameliorate the destabilizing effects of hydrostatic pressure.

TMAO is a potent protein stabilizer commonly found in the muscle tissues of marine fish species, and this stabilizer can alleviate the effects of hydrostatic pressure on protein stability and restore denatured proteins to their native structures [10,21]. In fish, TMAO is formed via the oxygenation of TMA by a hepatic enzyme, flavin-containing monooxygenase-3 (FMO3) [22]. However, TMA must be synthesized by the gut microbiota via two major pathways: TMA-lyase (CutC) in conjunction with its activator CutD utilizing choline as a substrate and a two-component Rieske-type oxygenase/reductase (CntA/B) acting on L-carnitine and its derivative gamma-butyrobetaine [23]. Consistent with expectations, the TMAO content in the YHS muscles (134 mmol/kg) is much higher than that in the large yellow croaker (43 mmol/kg) and zebrafish (0.07 mmol/kg; Fig 4A). Similar to Mariana hadal snailfish, five copies of the TMAO-generating gene (*fmo3*) were identified in the YHS genome, and four of them are tandem repeats (Fig 4B). The abundance of FMO3c transcripts in the YHS liver was greater than the abundance of all other FMO3 genes across all organs tested (Fig 4C), suggesting that FMO3c might be a major oxidase for TMAO production in YHS. An analysis of the gut microbiota of YHS showed that bacterial genera containing species carrying the *cutC* gene account for 8.8% of all microbes (*Escherichia/Shigella*, 2.42%; *Serratia*, 1.58%; *Vitreoscilla*, 1.02%; and *Acinetobacter*, 3.78%) and that bacterial genera containing species carrying the *cntA* gene account for 4.59% (*Escherichia/Shigella*; *Clostridium* sensu stricto 1, 0.08%; *Serratia*; and *Streptococcus*, 0.51%; Figs 4D and S6). The abundance of TMA, in conjunction with the five copies of the *fmo3* gene, might maintain a high level of TMAO in Yap hadal snailfish to improve protein stability under hadal conditions (Fig 4E).

### Sensory systems

**Fig 5:**
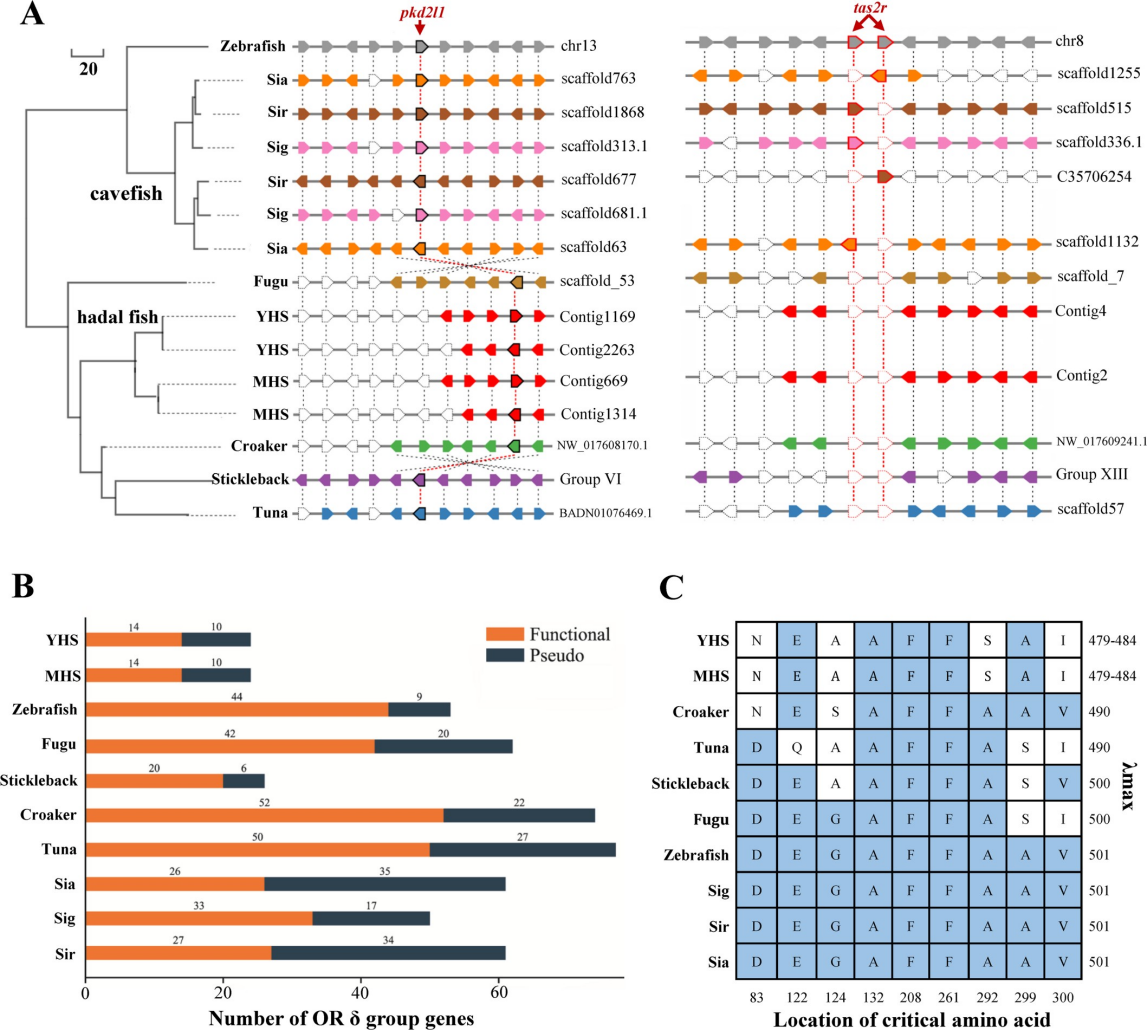
Genetic features of the unique sensory systems of Yap hadal snailfish. (A) Evolution and synteny of taste genes across several representative teleosts. The reference gene positions were based on the zebrafish genome. The white pentagons indicate lost genes. *Tas2r*: taste receptor type 2, *pkd2l1*: polycystic kidney disease 2-like 1. (B) Numbers of functional genes or pseudogenes encoding δ group olfactory receptors in various fish species. (C) Amino acid residues at the nine critical nine sites in rhodopsin across representative fish species and λmax values of rhodopsin in different fish species. YHS: Yap hadal snailfish, MHS: Mariana hadal snailfish, Sia: *Sinocyclocheilus anshuiensis*, Sig: *Sinocyclocheilus grahami*, Sir: *Sinocyclocheilus rhinocerous*.

Hadal environments are characterized by a low food supply and darkness, which are similar to the characteristics of underwater cave habitats [5,24]. We compared the genes associated with the sensory systems in hadal snailfishes to those of cave-restricted fishes (*Sinocyclocheilus anshuiensis*, *S*. *grahami*, and *S*. *rhinocerous*) and other shallow-water fish species to explore the genetic basis for their unique sensory characteristics. A syntenic analysis identified two copies of the sour taste receptor gene (polycystic kidney disease 2-like 1, *pkd2l1*) in both the YHS and Mariana hadal snailfish genomes (Fig 5A). In comparison, only one copy of *pkd2l1* was identified in other diploid teleosts, including zebrafish, stickleback, large yellow croaker, pufferfish (*Tetraodon nigroviridis*), and tuna (*Thunnus atlanticus*), whereas two copies were identified in tetraploid cavefishes (e.g., *Sinocyclocheilus* species) due to genome duplication [25]. We also found that the bitter taste gene (taste receptor type 2, *tas2r*) has been lost in the hadal snailfish genomes, even though reference genes have been found in zebrafish and *Sinocyclocheilus* cavefishes (Fig 5A). In addition, we predicted 40 olfactory receptor (OR) genes in the YHS genome, including 25 functional genes and 15 pseudogenes, similar to the results found for Mariana hadal snailfish (S20 Table). The genomes of hadal snailfishes have fewer olfactory receptor genes than the genomes of shallow-water teleosts, such as *Sinocyclocheilus* cavefishes (26–33 functional genes and 17–35 pseudogenes), pufferfish (44 functional genes and 54 pseudogenes), and zebrafish (102 functional genes and 35 pseudogenes; S20 Table). More specifically, only 14 functional genes encoding δ group olfactory receptors that are important for the perception of water-borne odorants [26], were identified in the YHS genome, and this number is far fewer than that found in zebrafish (Figs 5B and S7). The expansion of certain taste receptor genes and the massive loss of functional olfactory receptors in hadal snailfish genomes might be due to their specific dietary habits in hadal trenches. Trench communities are typically considered as nutrition-limited systems, and food resources are mainly supplemented by surface-derived carrion falls [1,5]. However, most of the sinking materials are consumed and intercepted by plankton and heterotrophic bacteria in shallower and bathyal waters. As the top predator in the hadal ecosystem, the snailfish possesses an inflated stomach that is typically filled with only one dominant crustacean species, *Hirondellea gigas* [27]. The relatively simple diet of the hadal snailfish and the limited food sources available to this species might have driven adaptive alterations in its taste sensation and olfaction. Therefore, the changes in the gene number of taste and olfaction receptors might facilitate the foraging of hadal snailfish in the nutrition-limited hadal environment.

Light in the hadal zone is extremely faint and has two primary natural sources: residual sunlight and bioluminescence [28]. Previous video observations have shown that the hadal snailfishes do not respond to strong light [19,29]. We therefore performed a comparative genomic analysis and found that the YHS had fewer copies of crystallin genes relative to those found in other sequenced teleosts (S8 Fig and S21 Table). Notably, the number of γ-crystallin genes in YHS was markedly lower than that in any of the shallow-water teleosts examined (S21 Table). The crystallins of the vertebrate eye lens are the predominant structural proteins that maintain the transparency and high refractive index of the lens, which enables the focusing of light on the retina [30]. The loss of crystallin genes suggests that the visual system of the YHS might have degenerated during life in the dark. We also identified two opsin genes, rhodopsin *rh1* and shortwave-sensitive *sws2*, in the YHS genome. The λmax of YHS rhodopsin is 479–484 nm, which is lower than the levels found in shallow-water teleosts (Fig 5C). Moreover, the same frameshift insertion in aralkylamine N-acetyltransferase 2 (*aanat2*), the critical gene for melatonin biosynthesis, was observed in both YHS (S9 Fig) and Mariana hadal snailfish [31], which led to the inactivation of AANAT2. This inactivation in the two hadal snailfishes may result in low levels of blood melatonin, which potentially reflects an adaptation to the darkness in hadal environments. Thus, Yap hadal snailfish might principally sense shortwave light, similar to fish with degenerated eyes, such as cavefish [25].

## Discussion

High hydrostatic pressures, low temperatures, and a scarce food supply are thought to be the major barriers to survival in the deep-sea environment, but a specialized fauna thrive in the hadal zone at depths exceeding 6000 m [32]. The most common vertebrate species in the hadal zone is snailfish, and the deepest snailfish recorded to date was captured from a depth exceeding 8,100 m [17]. Recently, the genetic basis and mechanisms of vertebrate adaptation to such an extreme environment have attracted more attention. Genomic analyses of a hadal snailfish from the Mariana Trench have revealed its adaptation to the extreme hydrostatic pressure, which possibly involve enhancing the cell membrane fluidity, transport protein activity, and protein stability [19]. Here, we sequenced and assembled a genome of another hadal snailfish from the Yap Trench. The final genome assembly is approximately 731.75 Mb, with a contig N50 of 0.75 Mb and a scaffold N50 of 1.26 Mb (Table 1), which are much higher than those found in the Mariana hadal snailfish genome; the genome assembly of Mariana hadal snailfish is 684 Mb, with a contig N50 of 0.34 Mb and a scaffold N50 of 0.42 Mb [19]. Thus, our assembly improved the genome quality of hadal snailfish. Coalescent species tree analysis recovered a sister relationship between Yap hadal snailfish and Mariana hadal snailfish, with a divergence time of approximately 0.92 Mya (Fig 2). In combination with their similar morphology and genome structure characteristics, Yap hadal snailfish and Mariana hadal snailfish might possess similar genetic features and adaptive mechanisms to the hadal ecosystem. Further genomic analyses revealed significant alterations in several gene families, such as taste receptors, olfactory receptors, and vision-related genes, in Yap hadal snailfish (Fig 5), and these alterations might provide the genetic basis for the adaptation of Yap hadal snailfish to the nutrition-limited and dark hadal environments.

DNA is vulnerable to high hydrostatic pressure, and hadal organisms must employ efficient DNA repair mechanisms to alleviate hydrostatic pressure-associated DNA damage [9,20]. Homologous recombination is a high-fidelity process that uses homologous DNA sequences as templates for the repair of damaged DNA [33]. RAD52 is an important member of the homologous recombination pathway, which promotes annealing between two complementary single-stranded DNA (ssDNA) molecules or between one ssDNA molecule and a complementary ssRNA molecule [34]. RAD9A, a DNA damage checkpoint protein, is essential for the DNA damage response [35]. Compared with shallow-water teleosts, substitutions with longer branched amino acids were detected in RAD52 (M68L and K167M) and RAD9A (V154F) of Yap hadal snailfish and Mariana hadal snailfish (Fig 3), which might contribute to the maintenance of the structure and function of these two proteins under ultrahigh hydrostatic pressure [2,36,37]. Similar circumstances were also observed in the hadal amphipod *Hirondellea gigas* [4], the hadal holothurian *Paelopatides* sp. [37] and the deep-sea fish *Aldrovandia affinis* [9]. In *H*. *gigas*, positive selection was observed in the replication factor A1 (RFA1) gene that is associated with DNA repair and maintenance of chromosomal stability [4]. Eight positively selected genes involved in DNA repair were identified in *Paelopatides* sp., including RAD9A [37]. Therefore, the amino acid substitutions in deep-sea organisms might reflect hydrostatic pressure-associated positive selection, suggesting that deep-sea species may share similar adaptive strategies. RAD51, a major eukaryotic homologous recombinase, plays a key role in homologous recombination by promoting the search for homologous double-stranded DNA (dsDNA) templates and repairing DNA double-strand breaks [38]. The expansion of *rad51* in Yap hadal snailfish might increase the DNA repair rates and thus facilitate the maintenance of DNA integrity under adverse environmental conditions. Therefore, the positive selection and expansion of genes needed for DNA repair imply that a stronger DNA repair capacity might be essential for the adaptation of hadal organisms to the high hydrostatic pressures in hadal environments.

Hydrostatic pressure affects protein folding and function [8]. Consequently, the organisms living in the hadal zone must use various mechanisms to maintain the stability of protein structures under elevated hydrostatic pressures [3]. TMAO is a physiologically strong protein stabilizer that plays a role in the stabilization of protein structures [10,21,39]. In this study, we found that the TMAO content in the muscle of Yap hadal snailfish was markedly higher than that in the muscle of shallow-water fish (Fig 4A), which is similar to the results found for other deep-sea fish [10,14,40]. TMAO is synthesized from TMA by host hepatic flavin monooxygenase 3 (FMO3) [22]. However, TMA cannot be synthesized in fish and is mainly produced by microbes in the fish gut [29]. An analysis of the gut microbiota showed that TMA-generating bacteria were abundant in the gut of Yap hadal snailfish (Fig 4D), suggesting that hadal snailfish might largely depend on these TMA-generating bacteria for their TMA supply. The synthesis of TMA by gut bacteria, in conjunction with the presence of five copies of *fmo3* in Yap hadal snailfish (Fig 4B), could facilitate the synthesis of TMAO in this species. The effective TMAO synthesis would help Yap hadal snailfish adapt to high hydrostatic pressure by improving protein stability under hadal conditions. To the best of our knowledge, this study constitutes the first demonstration of the role of the gut microbiota in the adaptation of hadal snailfish to high hydrostatic pressure. Though the abundance of TMA-generating bacteria in the gut was not analyzed, Mariana hadal snailfish also contain five copies of *fmo3* in their genomes and five putative promoters were predicted upstream of the *fmo3a* [19]. Thus, the enhancement of TMAO synthesis might represent a unique adaptation mechanism of hadal snailfishes to hadal environments, which would help the snailfishes live in the harsh hadal trenches.

In conclusion, we assembled a high-quality reference genome for Yap hadal snailfish. Comparative genomic analyses revealed significant alterations in several gene families associated with sensory systems and DNA repair in Yap hadal snailfish, providing the genetic basis for the adaptation of Yap hadal snailfish to hadal environments. The high levels of TMAO found in Yap hadal snailfish, which might be due to the presence of five copies of the TMAO-generating enzyme *fmo3* in the genome and the abundance of TMA-generating bacteria in the gut, might facilitate its adaptation to high hydrostatic pressure. Our results provide new insights into the molecular mechanisms underlying the adaptation of hadal organisms to the deep-sea environment. However, the exact functions of these positively selected and expanded genes in hadal snailfish adaptation require further investigation.

## Materials and methods

### Ethics statement

The studies were performed in strict accordance with the Regulations of the Administration of Affairs Concerning Experimental Animals established by the Fujian Provincial Department of Science and Technology. The animal experiments were approved by the Animal Care and Use Committee of the Fujian Agriculture and Forestry University (PZCASFAFU2019019). All efforts were made to minimize suffering.

### Sample collection and DNA/RNA extraction

One YHS individual was randomly selected for genomic analysis and genomic DNA was extracted from its liver tissue using the conventional sodium dodecyl sulfate (SDS) method [41]. The DNA quality was measured using a Bioanalyzer (Agilent Technologies, USA) and by agarose gel electrophoresis. Two mitochondrial fragments, the 16S rRNA gene (830 bp) and cytochrome c oxidase subunit I gene (COI, 645 bp), were amplified from the genomic DNA using polymerase chain reaction (PCR) using optimized primer pairs (16S rRNA-F: CTATTAATACCCCCAAATACCCC, 16S rRNA-R: CGATGTTTTTGGTAAACAGGCG; COI-F: TCAACCAACCACAAAGACATTGGCAC, COI-R: TAGACTTCTGGGTGGCCAAAGAATCA) [42]. The amplicons were sequenced using traditional Sanger sequencing. The 16S rRNA and COI gene sequences of four other hadal snailfishes (*P*. *swirei*, *Notoliparis kermadecensis*, *Rhodichthys regina*, and *Careproctus marginatus*) and two shallow-water snailfishes (*L*. *tanakae* and *Liparis ochotensis*) were retrieved from the GenBank database for comparison. Maximum likelihood (ML) trees were constructed based on each gene using MEGA 6.06 [43] with default parameters. Genetic distances were computed using the Kimura 2-parameter (K2P) algorithm. The rate variation among sites was modeled using a gamma distribution (shape parameter = 1), and all positions containing gaps and missing data were eliminated (i.e., the complete deletion option) [44]. Six tissues (eye, gut, liver, muscle, skin, and stomach) were then collected from the selected YHS for transcriptome sequencing.

### Library construction and sequencing

Genomic DNA was fragmented using a Covaris sonication system. Short-insert paired-end libraries (350 bp) were constructed according to Illumina’s protocol with end repair, poly-A tail base addition, sequencing-adaptor ligation, amplification, and purification. Paired-end sequencing was performed with an Illumina HiSeq X Ten platform (Illumina, USA). The Illumina raw reads were evaluated using FASTQC v0.11.6 [45] and filtered with fastp v0.20 [46] using the default parameters to produce the Illumina clean reads for subsequent analysis.

Simultaneously, the extracted genomic DNA was sheared using Megaruptor2 (Diagenode, Ougrée, Belgium), and then used to construct SMRT bell libraries via the ligation of universal hairpin adaptors onto double-stranded DNA fragments in accordance with the 20-kb preparation protocol (Pacific Biosciences, USA). The MagBead kit (Pacific Biosciences, USA) was used to remove adaptor dimers. The failed ligation products were removed using exonucleases. The sequencing primer was annealed to each SMRT bell template for subsequent sequencing with a PacBio Sequel instrument using Sequel Sequencing Kit 1.2.1 (Pacific Biosciences, USA). The PacBio polymerase reads were filtered using the RS_Subreads protocol with minimum length > 300 bp to produce the PacBio subreads for genome assembly.

### Genome-size estimation

We estimated the genome size of YHS based on the 17-mer frequency distribution of the 44.08-Gb Illumina clean reads using the following formula: genome size = (total number of 17-mers) / (position of the peak depth) [47].

### Genome assembly

All of the Illumina clean reads were subsequently split into small K-mers, and low-occurrence K-mers were removed. A de Bruijn graph was constructed using Platanus assembler v1.2.1 [48] with the following optimized parameters: input type = raw, genome size = 828360000, seed coverage = 50, and length cutoff pr = 10000. PacBio subreads longer than 300 bp were retained, corrected using Canu v1.5 [49], and assembled into an initial genome using Falcon v1.8.8 [50]. Pilon v1.22 [51] was used to polish the genome assembly using the clean reads. Finally, the PacBio subreads were scaffolded using SSPACE-LongRead v1-1 [52] with the default parameters. Gaps in the constructed scaffolds were filled based on the PacBio subreads using PBJelly v14.9.9 [53] with the default parameters.

### Assessment of the constructed genome assembly

Three approaches were integrated to evaluate the quality of the YHS genome assembly: read alignments, BUSCO (version 3.03) [54], and CEGMA (version 2.5) [55]. To assess the assembly based on read alignments, we mapped the Illumina clean reads onto scaffolds using BWA v0.6.2 [56] with the optimized parameters "-o 1 -i 15". Subsequently, to evaluate the completeness of the YHS genome assembly, we assessed the assembled scaffolds using BUSCO v3.03 (RRID: SCR_015008) [54] with the default parameters, against the conserved core genes in the Actinopterygii_odb9 database (4,584 orthologs). Finally, known genes in the genome assembly were aligned against a reliable set of 248 highly conserved proteins from a wide range of eukaryotes using CEGMA [55].

### Detection of repetitive sequences and noncoding RNAs

Repeat annotations were performed to clarify the genome assembly of YHS. We identified TEs using a combination of homology-based and *de novo* prediction methods. First, we used RepeatModeler v1.0.8 [57], LTR_FINDER v1.06 [58], and RepeatScout v1.0.5 [59] to build a *de novo* repeat library. Subsequently, we performed homology-based gene predictions using RepeatMasker [57] with the default parameters against Repbase and the *de novo* repeat libraries. Simultaneously, we used RepeatProteinMask [57] with the default parameters to identify repeated amino acid sequences. Tandem Repeats Finder [60] was used to identify tandem repeats with the following parameters: Match = 2, Mismatch = 7, Delta = 7, PM = 80, PI = 10, Minscore = 50, and MaxPeriod = 2000. After integration of all repeat annotation results using an in-house Perl script, we calculated the sequence divergence rate for each family of TEs. The genes associated with noncoding RNAs (ncRNAs) in the YHS genome, including microRNAs (miRNAs), ribosomal RNAs (rRNAs), small nuclear RNAs (snRNAs), and transfer RNAs (tRNAs), were predicted using Infernal v1.1.2 [61] based on alignments to the Rfam ncRNA database [62].

### Prediction of protein-coding genes

We used a combination of *de novo*, homology-based, and transcriptome-based methods for the prediction of protein-coding genes. We performed *de novo* prediction of the repeat-masked genome using Augustus v3.0.2 [63], Genescan v1.0 [64], Geneid v1.4 [65], GlimmerHMM v3.0.2 [66], and SNAP v2.0 [67]. To predict protein-coding genes based on homology, we obtained the longest available protein-coding sequences of eight representative vertebrate species from GenBank: human, large yellow croaker, spotted green pufferfish, three-spined stickleback, zebrafish, Nile tilapia (*Oreochromis niloticus*), fugu (*Takifugu rubripes*), and mouse (*Mus musculus*). These protein sequences were aligned to the assembled YHS genome using TBLASTN with an e-value < 1e-5, and the alignment results were integrated using SOLAR v0.96 [68,69]. GeneWise v2.2.0 was then used to predict gene models based on this alignment [70]. Protein-coding genes were also predicted based on the transcriptome data from six tissues (eye, intestine, liver, muscle, skin, and stomach) of YHS. We mapped the transcriptome data to the YHS genome assembly using TopHat v2.0.13 [71] and obtained gene structures using Cufflinks v2.1.1 [72]. In parallel, we assembled the transcriptome data using Trinity v2.1.1 [73] and obtained gene structures using PASA v2.3.3 [74]. Gene expression levels were determined based on fragments per kilobase of transcripts per million fragments mapped (FPKM) values using RNA-Seq by Expectation Maximization (RSEM) with the default settings [75]. The gene structures obtained using these three approaches were integrated with EVidenceModeler v1.1.1 [76] to yield a nonredundant gene set. Finally, we used PASA v2.3.3 [74] to adjust the gene models based on the assembled transcripts in order to obtain a final reference protein-coding gene set.

### Annotation of protein-coding genes

We functionally annotated the genes in the final protein-coding gene set to better understand their biological roles. First, we annotated the deduced protein sequences using InterProScan v4.7 [77] with the default settings, to identify motifs and domains. Then, Gene Ontology (GO) terms for each gene were assigned based on the corresponding InterPro descriptions. We then searched for the deduced protein sequences in the Kyoto Encyclopedia of Genes and Genomes (KEGG) database using the bidirectional best hit (BBH) method to identify associated pathways. Finally, we annotated the deduced protein sequences in the Swiss-Prot and TrEMBL databases with an e-value of 1e-5 for prediction of protein function.

### Genome evolution

Gene family clusters were determined using OrthoMCL v1.1 [78] based on 22 representative vertebrate genomes: Yap hadal snailfish, Mariana hadal snailfish, Tanaka’s snailfish, Atlantic salmon (*Salmo salar*), Atlantic cod (*Gadus morhua*), bicolor damselfish (*Stegastes partitus*), common carp (*Cyprinus carpio*), channel catfish (*Ictalurus punctatus*), electric eel (*Electrophorus electricus*), grass carp (*Ctenopharyngodon idella*), fugu, large yellow croaker, medaka (*Oryzias melastigma*), Mexican tetra (*Astyanax mexicanus*), Nile tilapia, spotted gar (*Lepisosteus oculatus*), spotted green pufferfish, Southern platyfish (*Xiphophorus maculatus*), stickleback, turquoise killifish (*Nothobranchius furzeri*), zebrafish, and human. For each species, we retained the longest transcript of each gene, and removed the genes encoding a protein consisting of less than 30 amino acids. We then identified all possible matches among the retained protein sequences through All-vs-All Blast with an e-value of 1e-7. Finally, we clustered the alignments into gene families using OrthoMCL [78] with an inflation index of 1.5.

The phylogenetic relationships among 22 representative vertebrates were then evaluated. First, the recombination for each single-copy gene shared across all representatives was tested with RDP4 [79] in two rounds using the methods RDP, CHIMERA, GENECONV, and MaxChi with Bonferroni correction (significance parameter set to 0.01). Second, the amino acid sequence of each remaining single-copy gene was separately aligned using MAFFT v7.237 [80] with the linsi package, and each served as a partitioned dataset. All of the aligned sequences were also concatenated and then served as the concatenated dataset. Third, the model for each dataset was suggested by ProtTest v3.4 [81]. A maximum likelihood (ML) phylogenetic tree of each dataset was then constructed based on the alignment using RAxML v8.2.12 [82]. Finally, a coalescent species tree was built with all of the passed dataset ML trees using ASTRAL v5.7.5 [83].

The divergence times among 22 representative vertebrates were estimated based on the constructed phylogenetic tree and the coding sequences using the MCMCtree module in PAML v4.8 [84], with the following primary parameters: clock = 2, alpha = 0.5, ncatG = 5, kappa_gamma = 6 2, alpha_gamma = 1 1, rgene_gamma = 2 20 1, sigma2_gamma = 1 10 1, burn-in = 4,000,000, sample-number = 100,000, and sample-frequency = 100. Two calibration points from TimeTree [85] were used as time priors: the divergence between human and zebrafish (413–443 Mya) [86] and the divergence between zebrafish and stickleback (206–252 Mya) [87].

Positive selection was inferred using the branch-site Ka/Ks test using the codeml module in PAML v4.8 [84] based on YHS (designated as the foreground phylogeny) and three shallow-water fish, including stickleback, large yellow croaker, and zebrafish (designated as the background phylogeny). The phylogenetic relationship across these four species was reconstructed with the concatenated sequences as described above. ParaAT v2.0 [88] with a “-g” parameter was used to align the coding DNA sequences of each ortholog according to its amino acid sequence alignment. An alternative branch site model (Model = 2, NSsites = 2, and fix_omega = 0) and a neutral branch site model (Model = 2, NSsites = 2, fix_omega = 1 and omega = 1) were configured. *P-*values were first computed by a chi-squared test [89] and then corrected by a multiple testing correction [90]. Genes with Bayesian Empirical Bayes (BEB) sites exceeding 90% and corrected *P-*values lower than 0.1 were considered as positively selected genes. The InterProScan annotation results of positively selected genes were used to obtain the GO term assignments with the BLAST2GO v3.1.3 (e-value <1e-6, *P*-value < 0.05) [91].

### Contraction and expansion of gene families

To examine the evolutionary history of the identified gene families, we estimated their expansion and contraction in the YHS genome and then identified those that were substantially altered across Mariana hadal snailfish, Tanaka’s snailfish, the other 18 other shallow-water teleost species and human (serving as the outgroup). The expanded and contracted gene families were identified using CAFÉ v4.2.1 [92] after the removal of those gene families with more than 200 copies in a single species but fewer than two copies in any other species. By comparing each branch to its ancestral branch, we calculated *P-*values using Fisher’s exact test and then adjusted these *P-*values based on the false discovery rate [93]. Gene families with adjusted *P-*values less than 0.05 were considered to have undergone significant contraction or expansion during evolution. The expanded domains in the YHS genome were identified using Pfam [94] with an e-value <1e-5.

### Identification of the sensory genes in teleost genomes

We downloaded the sequences of zebrafish taste receptor genes (sour, sweet, umami, bitter, and salty), olfactory receptor (OR) genes, visual opsin genes, and crystallin genes from the GenBank or Ensembl databases. We used tBlastn v2.26 [95] to search these genes against the genomes of YHS, Mariana hadal snailfish, Fugu, stickleback, large yellow croaker, tuna, and three cave-restricted fishes (*Sinocyclocheilus anshuiensis*, *S*. *grahami*, and *S*. *rhinocerous*) with an e-value < 1e-5. We also collected the upstream and downstream genes to finish the syntenic analysis. The protein sequences were aligned using the einsi package in MAFFT v7.237 [80], and phylogenetic trees were constructed using FastTree v2.1.10 [96]. The rhodopsin λmax values were estimated according to a previous study [97].

### Determination of the TMAO content by LC-MS/MS

The TMAO contents (per kilogram of wet tissue) in the muscle tissues of Yap hadal snailfish, large yellow croaker (a shallow-sea fish), and zebrafish were measured by high-performance liquid chromatography and mass spectrometry (LC-MS/MS). In brief, 50 mg of muscle sample was mixed with methanol containing 50 ng/mL deuterium-labeled methyl d9-TMAO (d9-TMAO) as an internal standard. The mixture was homogenized for 2 min and centrifuged at 4°C and 13000 rpm for 10 min. The supernatant (2 μL) was then injected into an Agilent 1290 Infinity II UHPLC System (Santa Clara, CA, USA) coupled to Agilent 6470 Triple Quadruple MS/MS. Analytes were separated on an XBridge BEH HILIC Column (100 mm x 2.1 mm, particle size of 2.5 μm) at room temperature. Mobile phase A was 0.15% formic acid and 10 mM ammonium acetate in water; mobile phase B was 100% acetonitrile. The mobile phase was run isocratically at a flow rate of 0.35 mL/min based on the following program: 0–1 min, 98% B; 1–6 min, 98–90% B; 6–7 min, 90% B; 7–10 min, 90–85% B; 10–12 min, 85–70% B; and 12–13 min, 70–60% B. The compounds were ionized by electrospray ionization operated in the positive mode. The capillary temperature was set to 300°C, the gas source temperature was 350°C, and the ESI voltage was 3500 V. The ion pairs used for the qualitative analysis were m/z 76–58 for TMAO and m/z 85–66 for d9-TMAO. The concentration of TMAO was calculated using QQQ quantitative analysis software (Agilent, USA).

### Cloning and classification of 16S rRNA gene sequences

Total DNA was also extracted from the YHS gut for bacterial 16S rRNA gene sequencing. The V3–V4 region of the microbial 16S rRNA gene was amplified using universal primers (341F: CCTACGGGNGGCWGCAG; 806R: GGACTACHVGGGTATCTAAT). Purified amplicons were pooled in equimolar volumes and used for paired-end sequencing (2 × 250) with an Illumina platform (HiSeq 2500) following standard protocols. After quality control, the paired-end clean reads were merged using FLASH (v1.2.11) [98] and filtered with QIIME (v1.9.1) [99]. Taxonomic assignments were performed based on the SILVA database (www.arb-silva.de/) using the RDP classifier v2.2 [100] with the default settings.

## Supporting information

S1 Fig Phylogenetic relationships among the hadal snailfish (YHS) from the Yap Trench and other snailfish species. Maximum likelihood (ML) trees were constructed based on 16S rRNA (A) and cytochrome c oxidase subunit I (COI; B) genes. The 16S rRNA and COI gene sequences of the two Yap hadal snailfish specimens were consistent. ML bootstrap support values (> 50%) are shown. Branches with < 50% support have been collapsed.
(PDF)

S2 Fig Comparison of the gene sets obtained using three prediction methods. Genes in the Yap hadal snailfish genome were predicted using a combination of three approaches: *de novo*, homolog-based, and transcriptome-based methods. More than 98% of the 23,853 predicted genes were supported by at least two methods.
(PDF)

S3 Fig Comparison of gene structure characteristics among Yap hadal snailfish and other vertebrates. The lines with different colors represent different species.
(PDF)

S4 Fig BUSCO completeness assessment of 21 teleost species. The genome completeness of 21 teleost species was estimated using BUSCO v3.03 against the Actinopterygii_odb9 database. YHS: Yap hadal snailfish, MHS: Mariana hadal snailfish.
(PDF)

S5 Fig Gene family characteristics of the genomes of Yap hadal snailfish (YHS) and other representative vertebrates. Gene family clusters were determined using OrthoMCL v 1.1. For each species, the longest transcript of each gene was retained, whereas the genes encoding a protein consisting of less than 30 amino acids were removed.
(PDF)

S6 Fig Tag distribution in the Yap hadal snailfish gut at different levels of classification. (PDF)

S7 Fig Phylogenetic tree of the functional δ group olfactory receptor genes of Yap hadal snailfish (YHS) and zebrafish. (PDF)

S8 Fig Phylogenetic tree of the γ-crystallin genes of Yap hadal snailfish (YHS) and zebrafish. (PDF)

S9 Fig Frameshift mutation in the ANNAT gene of Yap hadal snailfish (YHS). A frameshift mutation was identified in the YHS genome that led to a severe mutation in the open reading frame (ORF) of the ANNAT gene. The translated protein according to the mutated ORF is completely distinct from that of Medaka ANNAT.
(PDF)

S1 Table Summary of sequencing data for Yap hadal snailfish (YHS). (PDF)

S2 Table Estimation of Yap hadal snailfish genome size (Kmer = 17). (PDF)

S3 Table Summary of the genome assembly of Yap hadal snailfish. (PDF)

S4 Table Statistics of the genome reads coverage. (PDF)

S5 Table The CEGMA evaluation of genome assembly. (PDF)

S6 Table The BUSCO evaluation of genome assembly. (PDF)

S7 Table Statistics of the genome base content. (PDF)

S8 Table SNP results of Yap hadal snailfish genome. (PDF)

S9 Table Summary of repeats in Yap hadal snailfish genome. (PDF)

S10 Table Transposable elements in Yap hadal snailfish genome. (PDF)

S11 Table Statistics of noncoding RNA of Yap hadal snailfish genome. (PDF)

S12 Table Prediction of gene structure in Yap hadal snailfish genome. (PDF)

S13 Table Information for the RNA-seq data from different Yap hadal snailfish tissues. (PDF)

S14 Table Gene structures of Yap hadal snailfish and other teleost genomes. (PDF)

S15 Table Functional annotation of genes in Yap hadal snailfish genome. (PDF)

S16 Table The accession numbers for genome assemblies used in this study. (PDF)

S17 Table GO enrichment (Level 4) of positively selected genes from the genome assembly of Yap hadal snailfish. (PDF)

S18 Table The positively selected genes involved in DNA repair from Yap hadal snailfish. (PDF)

S19 Table List of Pfam domains with more copies in Yap hadal snailfish than in other species. (PDF)

S20 Table Numbers of olfactory receptor (OR) genes in examined fish species. (PDF)

S21 Table The copy number of crystalline genes in Yap hadal snailfish and other species. (PDF)
