# Supplementary material for: Whole genome sequencing of a snailfish from the Yap Trench (~7,000 m) clarifies the molecular mechanisms underlying adaptation to the deep sea
Source: PLoS Genet. 2021 May 13;17(5):e1009530. doi: 10.1371/journal.pgen.1009530 (PMC8118300; doi:10.1371/journal.pgen.1009530)
Supplement: S1 Fig — Maximum likelihood (ML) trees were constructed based on 16S rRNA (A) and cytochrome c oxidase subunit I (COI; B) genes. The 16S rRNA and COI gene sequences of the two Yap hadal snailfish specimens were consistent. ML bootstrap support values (> 50%) are shown. Branches with < 50% support have been collapsed. (PDF) [file pgen.1009530.s001.pdf]

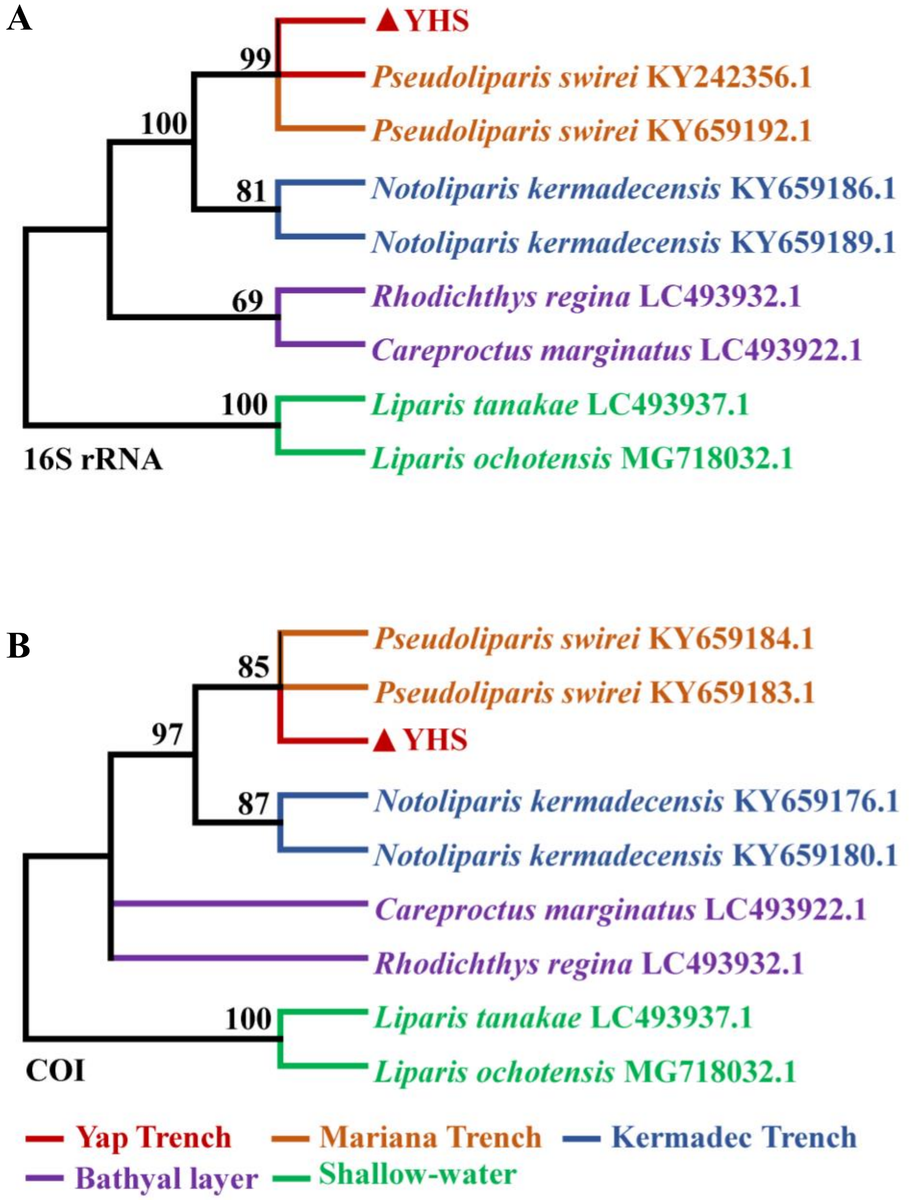

S1 Fig. Phylogenetic relationships among the hadal snailfish (YHS) from the Yap trench and other snailfish species. Maximum likelihood (ML) trees were constructed based on 16S rRNA (A) and cytochrome c oxidase subunit I (COI; B) genes. The 16S rRNA and COI gene sequences of the two Yap hadal snailfish specimens were consistent. ML bootstrap support values (> 50%) are shown. Branches with < 50% support have been collapsed.
