# Supplementary material for: Whole genome sequencing of a snailfish from the Yap Trench (~7,000 m) clarifies the molecular mechanisms underlying adaptation to the deep sea
Source: PLoS Genet. 2021 May 13;17(5):e1009530. doi: 10.1371/journal.pgen.1009530 (PMC8118300; doi:10.1371/journal.pgen.1009530)
Supplement: S2 Fig — Genes in the Yap hadal snailfish genome were predicted using a combination of three approaches: de novo, homolog-based, and transcriptome-based methods. More than 98% of the 23,853 predicted genes were supported by at least two methods. (PDF) [file pgen.1009530.s002.pdf]

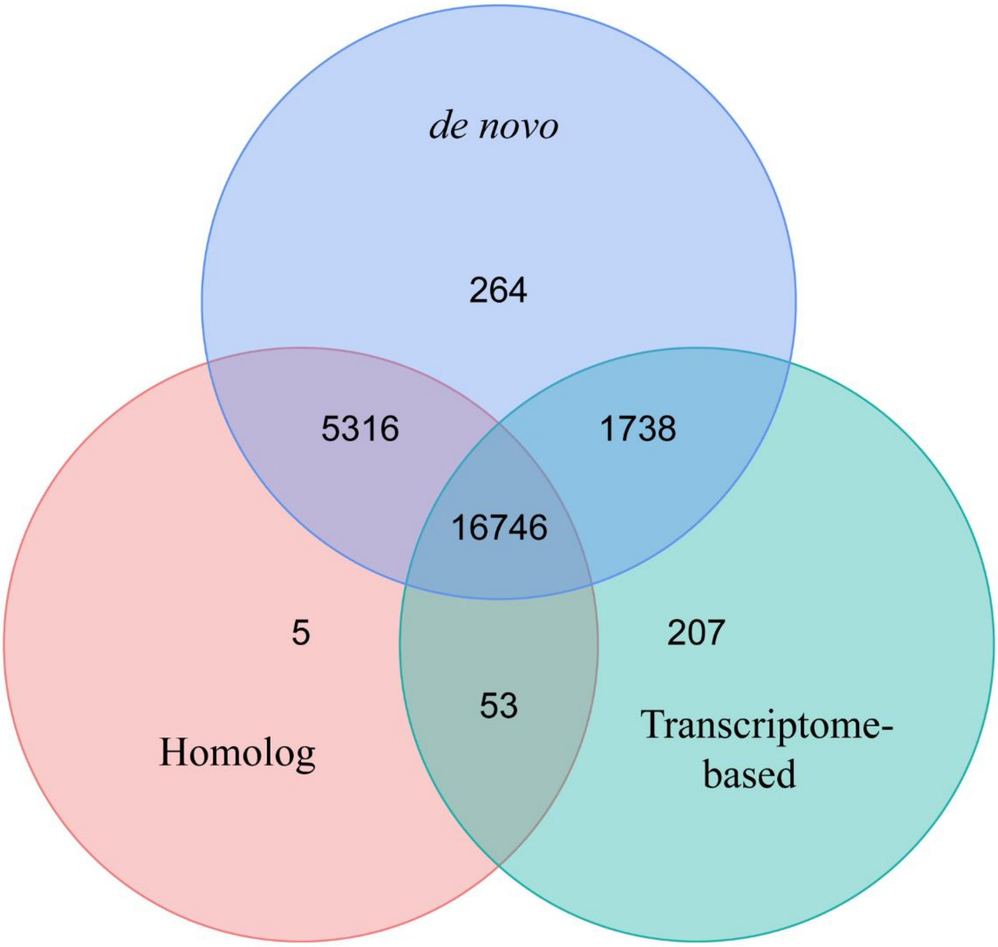

S2 Fig. Comparison of the gene sets obtained using three prediction methods. Genes in the Yap hadal snailfish genome were predicted using a combination of three approaches: *de novo*, homolog-based, and transcriptome-based methods. More than 98% of the 23,853 predicted genes were supported by at least two methods.
