# Supplementary material for: Whole genome sequencing of a snailfish from the Yap Trench (~7,000 m) clarifies the molecular mechanisms underlying adaptation to the deep sea
Source: PLoS Genet. 2021 May 13;17(5):e1009530. doi: 10.1371/journal.pgen.1009530 (PMC8118300; doi:10.1371/journal.pgen.1009530)
Supplement: S3 Fig — The lines with different colors represent different species. (PDF) [file pgen.1009530.s003.pdf]

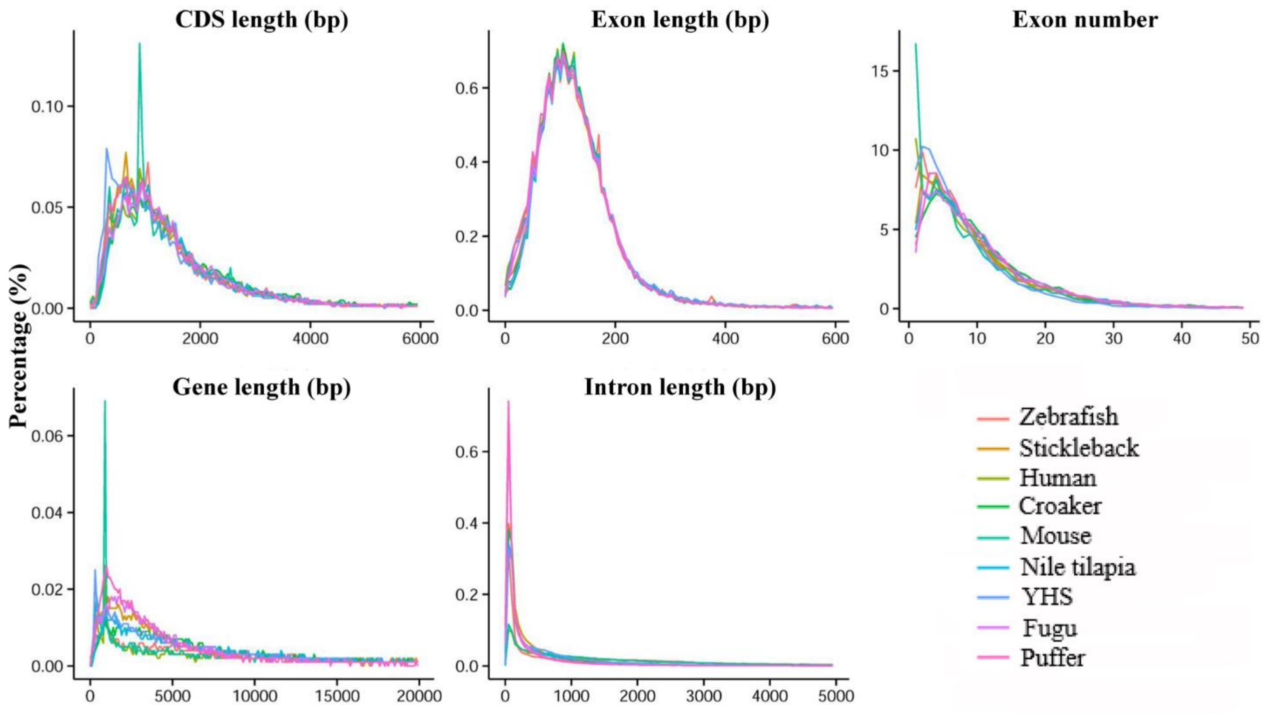

S3 Fig. Comparison of gene structure characteristics among Yap hadal snailfish (YHS) and other vertebrates. The lines with different colors represent different species.
