# Supplementary material for: Whole genome sequencing of a snailfish from the Yap Trench (~7,000 m) clarifies the molecular mechanisms underlying adaptation to the deep sea
Source: PLoS Genet. 2021 May 13;17(5):e1009530. doi: 10.1371/journal.pgen.1009530 (PMC8118300; doi:10.1371/journal.pgen.1009530)
Supplement: S4 Fig — The genome completeness of 21 teleost species was estimated using BUSCO v3.03 against the Actinopterygii_odb9 database. YHS: Yap hadal snailfish, MHS: Mariana hadal snailfish. (PDF) [file pgen.1009530.s004.pdf]

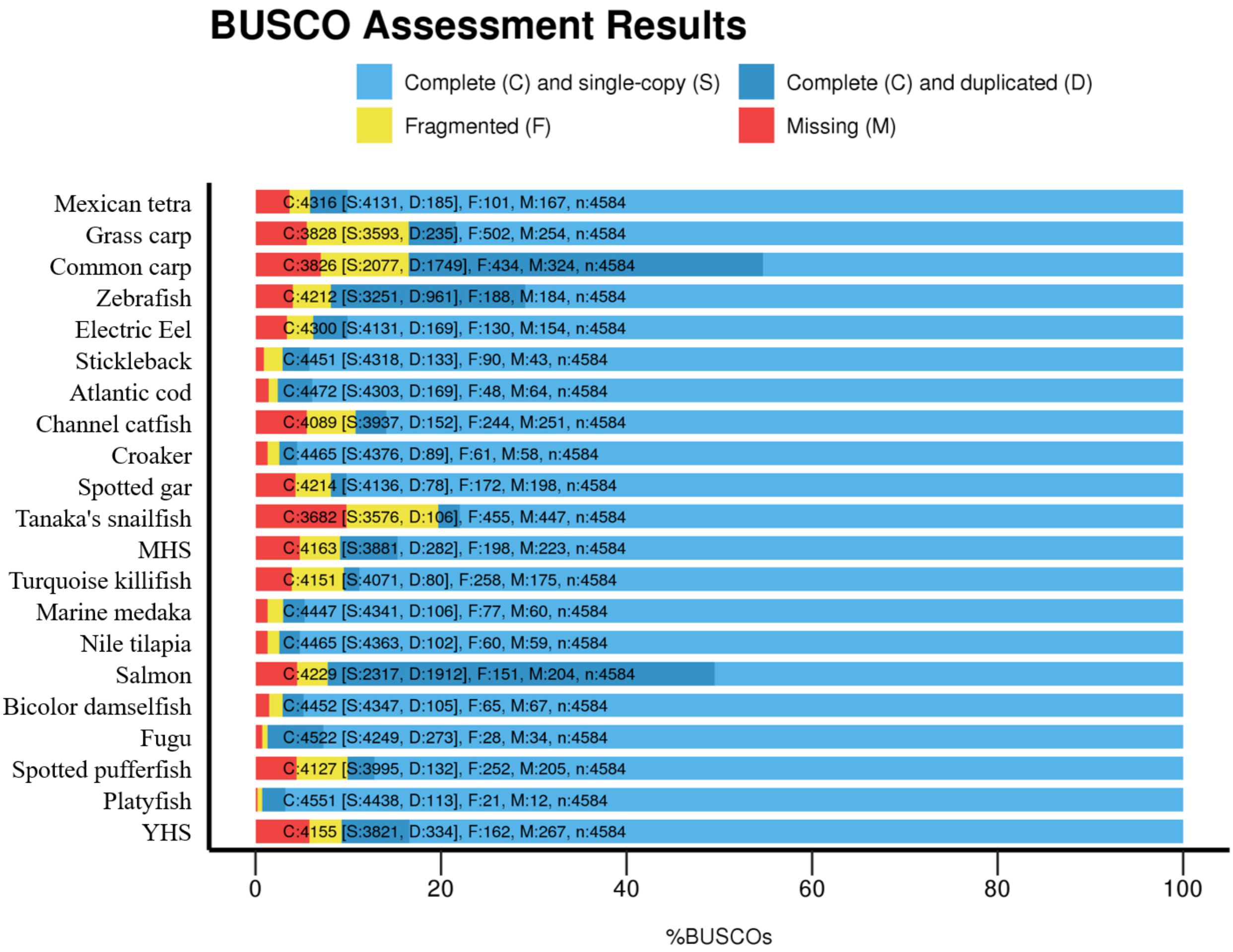

S4 Fig. BUSCO completeness assessment of 21 teleost species. The genome completeness of 21 teleost species was estimated using BUSCO v3.03 against the Actinopterygii_odb9 database. YHS: Yap hadal snailfish, MHS: Mariana hadal snailfish.
