# Supplementary material for: Whole genome sequencing of a snailfish from the Yap Trench (~7,000 m) clarifies the molecular mechanisms underlying adaptation to the deep sea
Source: PLoS Genet. 2021 May 13;17(5):e1009530. doi: 10.1371/journal.pgen.1009530 (PMC8118300; doi:10.1371/journal.pgen.1009530)
Supplement: S5 Fig — Gene family clusters were determined using OrthoMCL v 1.1. For each species, the longest transcript of each gene was retained, whereas the genes encoding a protein consisting of less than 30 amino acids were removed. (PDF) [file pgen.1009530.s005.pdf]

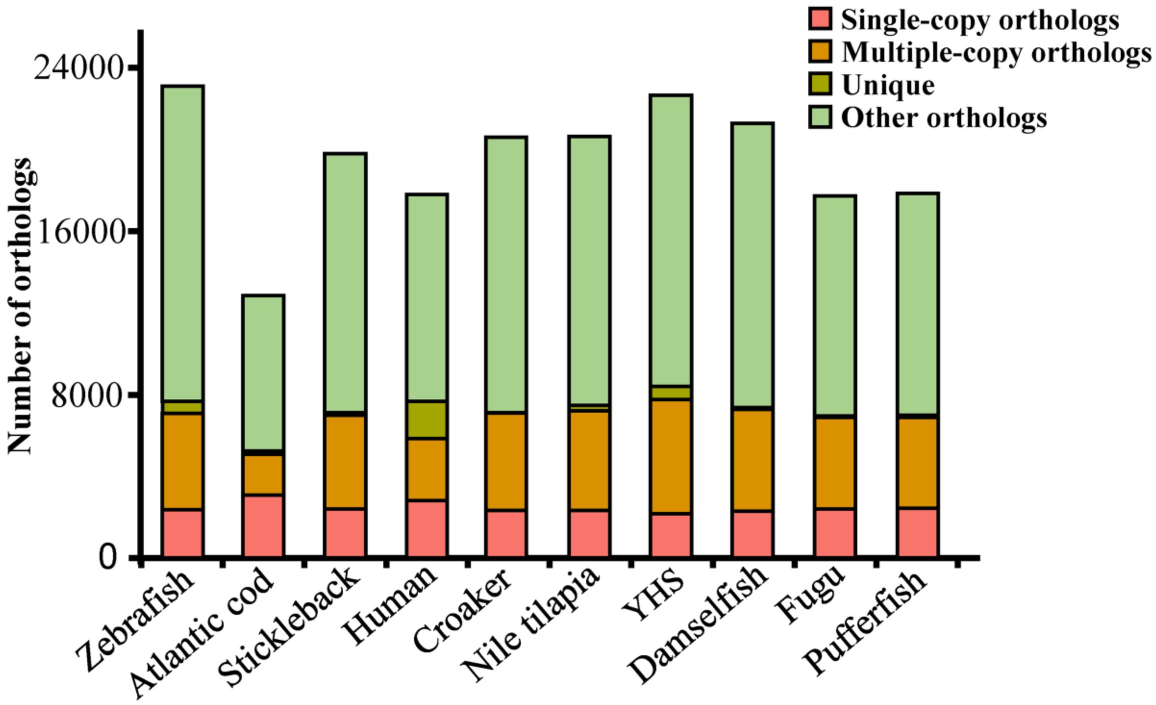


**S5 Fig. Gene family characteristics in the genomes of Yap hadal snailfish (YHS) and other representative vertebrates.** Gene family clusters were determined by OrthoMCL v 1.1. For each species, the longest transcript for each gene was retained, whereas the genes encoding a protein consisting of less than 30 amino acids were removed.
