# Supplementary figures and images for: Whole genome sequencing of a snailfish from the Yap Trench (~7,000 m) clarifies the molecular mechanisms underlying adaptation to the deep sea
Source: PLoS Genet. 2021 May 13;17(5):e1009530. doi: 10.1371/journal.pgen.1009530 (PMC8118300; doi:10.1371/journal.pgen.1009530)

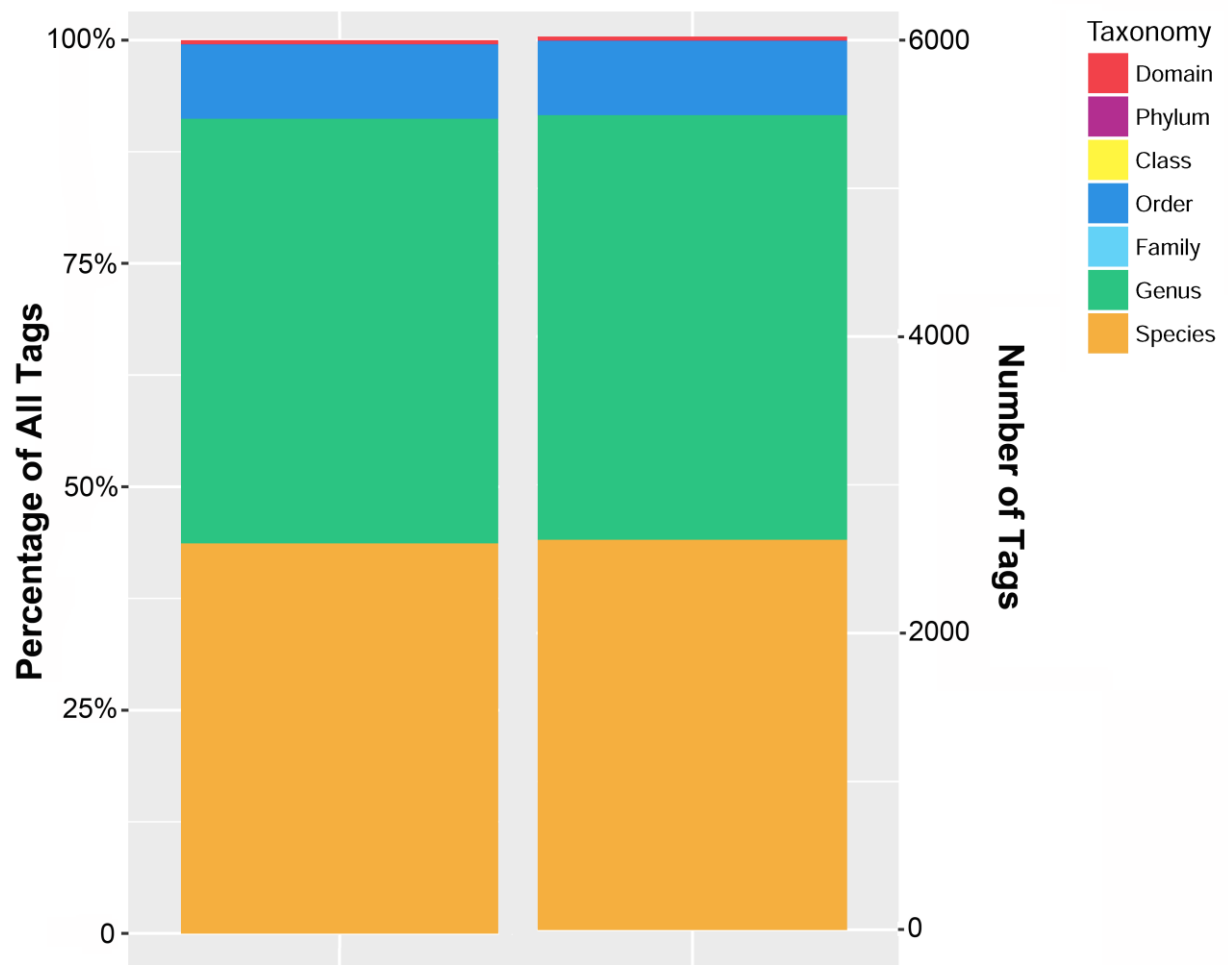

**S6 Fig. Tag distribution in the Yap hadal snailfish gut at different levels of classification.**

Supplement: S6 Fig — (PDF) [file pgen.1009530.s006.pdf]

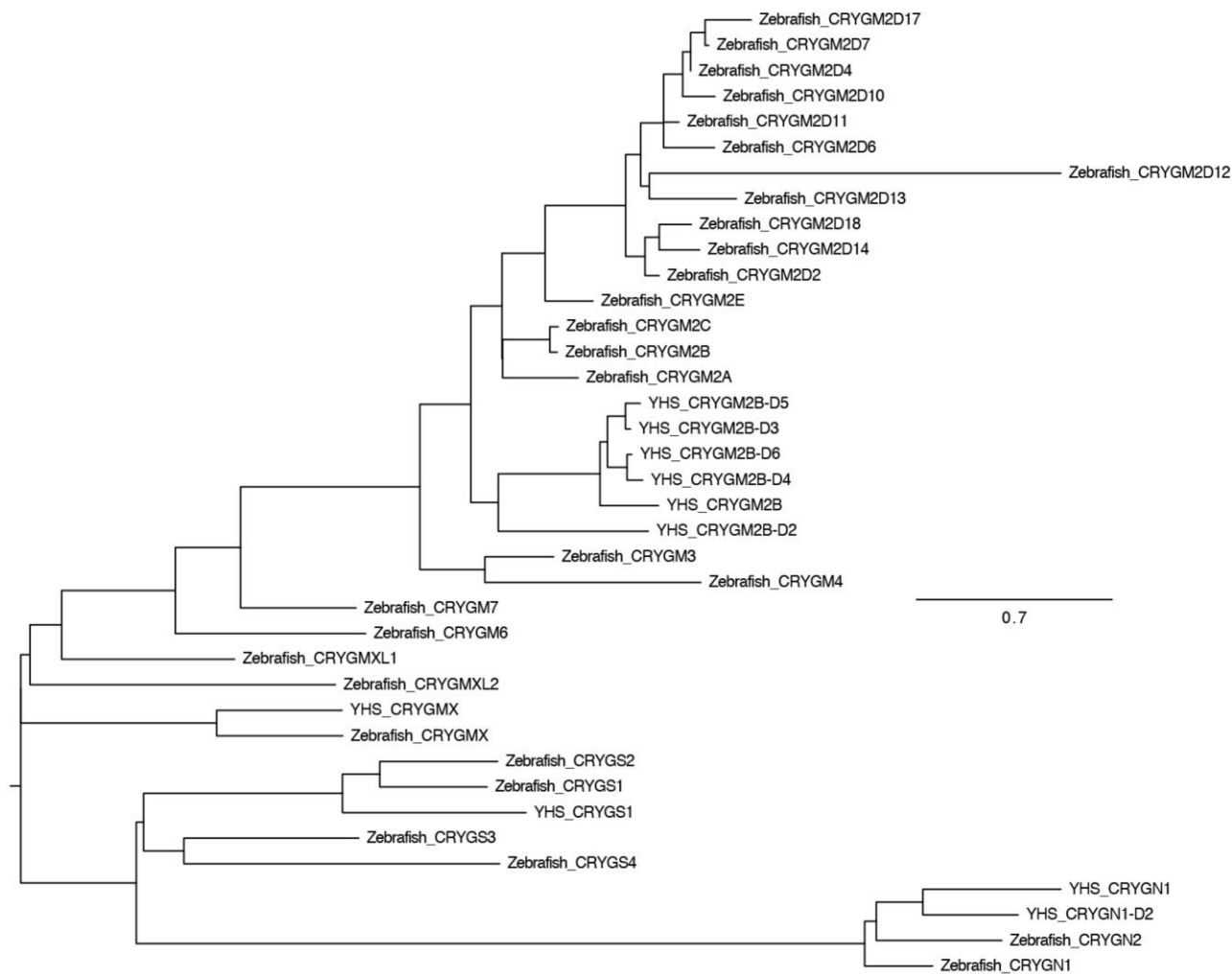

**S8 Fig. Phylogenetic tree of the  $\gamma$ -crystallin genes of Yap hadal snailfish (YHS) and zebrafish.**

Supplement: S8 Fig — (PDF) [file pgen.1009530.s008.pdf]
