# Supplementary material for: Whole genome sequencing of a snailfish from the Yap Trench (~7,000 m) clarifies the molecular mechanisms underlying adaptation to the deep sea
Source: PLoS Genet. 2021 May 13;17(5):e1009530. doi: 10.1371/journal.pgen.1009530 (PMC8118300; doi:10.1371/journal.pgen.1009530)
Supplement: S7 Fig — (PDF) [file pgen.1009530.s007.pdf]

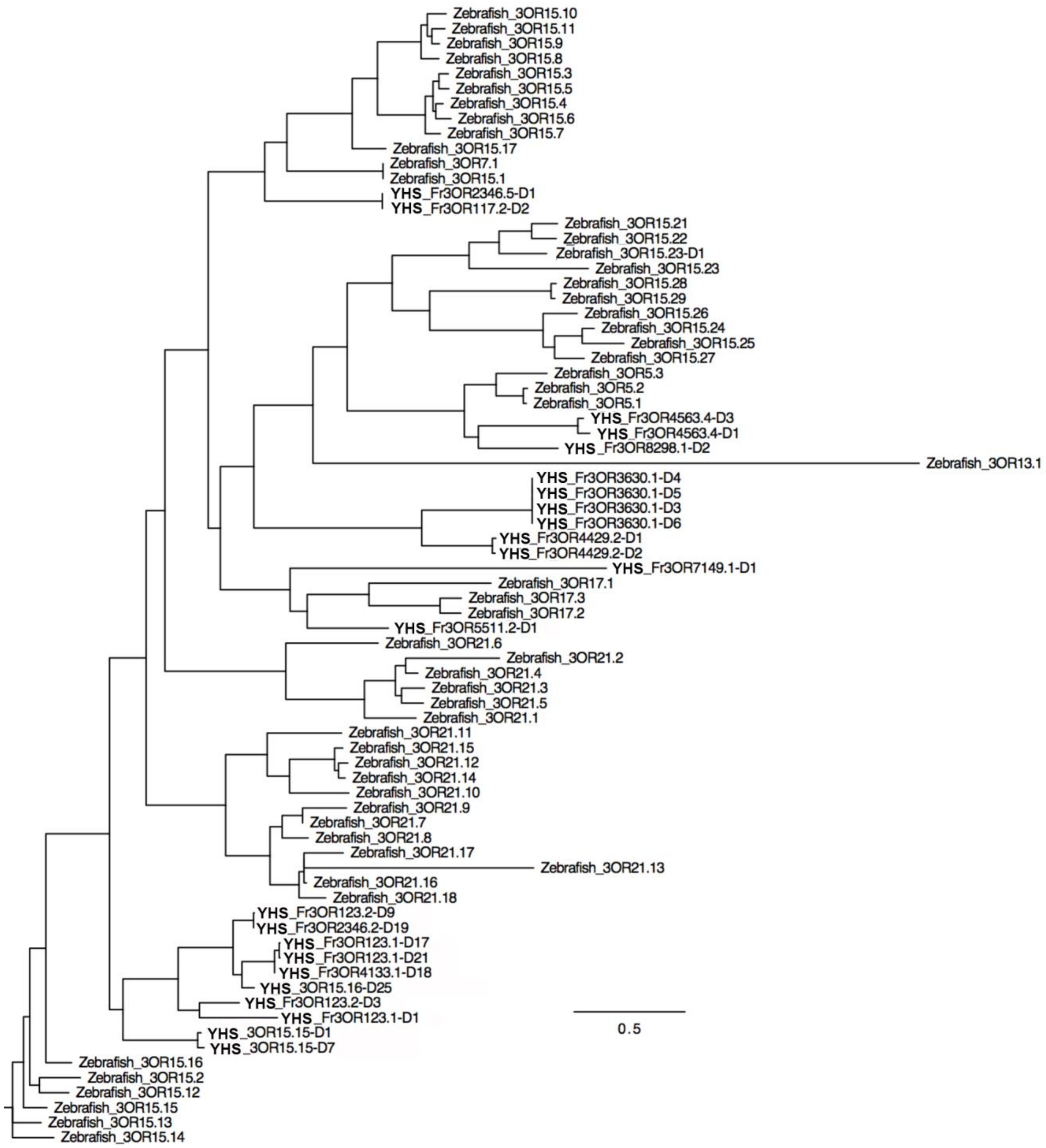

S7 Fig. Phylogenetic tree of the functional δ group olfactory receptor genes of Yap hadal snailfish (YHS) and zebrafish.
