# Supplementary material for: Whole genome sequencing of a snailfish from the Yap Trench (~7,000 m) clarifies the molecular mechanisms underlying adaptation to the deep sea
Source: PLoS Genet. 2021 May 13;17(5):e1009530. doi: 10.1371/journal.pgen.1009530 (PMC8118300; doi:10.1371/journal.pgen.1009530)
Supplement: S1 Table — (PDF) [file pgen.1009530.s010.pdf]

S1 Table. Summary of sequencing data for Yap hadal snailfish (YHS).

Platform	Illumina Hiseq X Ten		PacBio Sequel RSII	
Insert size (bp)	350 bp		9 kb	
	Raw data	Clean data	Polymerase reads	Subreads
Number of bases (Gb)	48.83	44.08	81.02	80.87
Number of reads	162,779,110	146,943,996	6,709,838	9,991,427
Mean reads length (bp)	150	150	12,075	8,093
Reads N50 (kp)	-	-	20.58	12,688
Sequence coverage (×)	59.87	54.05	99.34	99.15
SRA accession	PRJNA512070 (SRR8510169~ SRR8510172)		PRJNA512070 (SRR8510163~ SRR8510168; SRR8929648~ SRR8929652; SRR8929654; SRR8929657~ SRR8929660)	
