# Supplementary material for: Whole genome sequencing of a snailfish from the Yap Trench (~7,000 m) clarifies the molecular mechanisms underlying adaptation to the deep sea
Source: PLoS Genet. 2021 May 13;17(5):e1009530. doi: 10.1371/journal.pgen.1009530 (PMC8118300; doi:10.1371/journal.pgen.1009530)
Supplement: S2 Table — (PDF) [file pgen.1009530.s011.pdf]

S2 Table. Estimation of the Yap hadal snailfish genome size (Kmer = 17).

K-mer	K-mer number	K-mer Depth	Genome Size （Mb）	Heterozygous Ratio (%)
17	39,380,990,928	47	815.59	0.61
