# Supplementary material for: Whole genome sequencing of a snailfish from the Yap Trench (~7,000 m) clarifies the molecular mechanisms underlying adaptation to the deep sea
Source: PLoS Genet. 2021 May 13;17(5):e1009530. doi: 10.1371/journal.pgen.1009530 (PMC8118300; doi:10.1371/journal.pgen.1009530)
Supplement: S3 Table — (PDF) [file pgen.1009530.s012.pdf]

S3 Table. Summary of the genome assembly of Yap hadal snailfish.

Sample ID	Length		Number	
	Contig (bp)	Scaffold (bp)	Contig	Scaffold
Total	728,608,360	731,750,508	3,803	1,271
Max	8,287,565	8,357,545	-	-
Number>=2000	-	-	3,764	1,248
N50	753,890	1,256,700	213	159
N60	451,277	889,758	337	229
N70	247,462	646,228	556	326
N80	143,792	431,389	943	467
N90	73,673	259,655	1,650	686
