# Supplementary material for: Whole genome sequencing of a snailfish from the Yap Trench (~7,000 m) clarifies the molecular mechanisms underlying adaptation to the deep sea
Source: PLoS Genet. 2021 May 13;17(5):e1009530. doi: 10.1371/journal.pgen.1009530 (PMC8118300; doi:10.1371/journal.pgen.1009530)
Supplement: S4 Table — (PDF) [file pgen.1009530.s013.pdf]

S4 Table. Statistics of the genome reads coverage.

**Reads**	Mapping rate	95.7%
**Genome**	Average sequencing depth	59.74 ×
	Coverage	96.17%
	Coverage at least 4×	95.08%
	Coverage at least 10×	93.82%
	Coverage at least 20×	90.76%
