# Supplementary material for: Whole genome sequencing of a snailfish from the Yap Trench (~7,000 m) clarifies the molecular mechanisms underlying adaptation to the deep sea
Source: PLoS Genet. 2021 May 13;17(5):e1009530. doi: 10.1371/journal.pgen.1009530 (PMC8118300; doi:10.1371/journal.pgen.1009530)
Supplement: S5 Table — (PDF) [file pgen.1009530.s014.pdf]

S5 Table. CEGMA evaluation of the genome assembly.

Species	Complete		Complete + Partial	
	Prots	Completeness (%)	Prots	Completeness (%)
Yap hadal snailfish	222	89.52	228	91.94
