# Supplementary material for: Whole genome sequencing of a snailfish from the Yap Trench (~7,000 m) clarifies the molecular mechanisms underlying adaptation to the deep sea
Source: PLoS Genet. 2021 May 13;17(5):e1009530. doi: 10.1371/journal.pgen.1009530 (PMC8118300; doi:10.1371/journal.pgen.1009530)
Supplement: S6 Table — (PDF) [file pgen.1009530.s015.pdf]

S6 Table. BUSCO evaluation of the genome assembly.

BUSCO categories	Percentage
Complete	90.3%
Complete single-copy	83.3%
Complete duplicate	7.0%
Fragmented	4.3%
Missing BUSCOs	5.4%
Total BUSCO groups searched	2586
