# Supplementary material for: Whole genome sequencing of a snailfish from the Yap Trench (~7,000 m) clarifies the molecular mechanisms underlying adaptation to the deep sea
Source: PLoS Genet. 2021 May 13;17(5):e1009530. doi: 10.1371/journal.pgen.1009530 (PMC8118300; doi:10.1371/journal.pgen.1009530)
Supplement: S7 Table — (PDF) [file pgen.1009530.s016.pdf]

S7 Table. Statistics of the genome base content.

Base	Number	Percentage (%)
A	204,202,736	27.91
T	204,436,986	27.94
C	159,979,576	21.86
G	159,989,266	21.86
N	3,142,148	0.43
Total	731,750,712	-
GC	319,968,842	43.92
