# Supplementary material for: Whole genome sequencing of a snailfish from the Yap Trench (~7,000 m) clarifies the molecular mechanisms underlying adaptation to the deep sea
Source: PLoS Genet. 2021 May 13;17(5):e1009530. doi: 10.1371/journal.pgen.1009530 (PMC8118300; doi:10.1371/journal.pgen.1009530)
Supplement: S8 Table — (PDF) [file pgen.1009530.s017.pdf]

S8 Table. SNP results of the Yap hadal snailfish genome.

Categories	Number	Percentage (%)
All SNP	1,392,120	0.2100
Heterozygosis SNP	1,377,731	0.2078
Homology SNP	14,389	0.0022
