# Supplementary material for: Whole genome sequencing of a snailfish from the Yap Trench (~7,000 m) clarifies the molecular mechanisms underlying adaptation to the deep sea
Source: PLoS Genet. 2021 May 13;17(5):e1009530. doi: 10.1371/journal.pgen.1009530 (PMC8118300; doi:10.1371/journal.pgen.1009530)
Supplement: S9 Table — (PDF) [file pgen.1009530.s018.pdf]

S9 Table. Summary of repeats in the Yap hadal snailfish genome.

Type	Repeat Size (bp)	Percentage (%)
TRF	117,024,159	15.99
RepeatMasker	349,596,427	47.78
ProteinMask	69,005,392	9.43
Total	392,262,661	53.61
