# Supplementary material for: Whole genome sequencing of a snailfish from the Yap Trench (~7,000 m) clarifies the molecular mechanisms underlying adaptation to the deep sea
Source: PLoS Genet. 2021 May 13;17(5):e1009530. doi: 10.1371/journal.pgen.1009530 (PMC8118300; doi:10.1371/journal.pgen.1009530)
Supplement: S10 Table — (PDF) [file pgen.1009530.s019.pdf]

**S10 Table. Transposable elements in the Yap hadal snailfish genome.**

| Type | RepeatMasker (repbase + *de novo*) | | TE proteins | | Combined TEs | |
|---|---|---|---|---|---|---|
| | Length (bp) | % in genome | Length (bp) | In genome (%) | Length (bp) | % in genome |
| DNA | 95,523,096 | 13.06 | 7,104,819 | 0.97 | 99,044,095 | 13.54 |
| LINE | 162,614,128 | 22.22 | 47,065,842 | 6.43 | 175,457,523 | 23.98 |
| SINE | 2,920,405 | 0.40 | 0 | 0 | 2,920,405 | 0.40 |
| LTR | 93,784,468 | 12.84 | 14,978,264 | 2.05 | 95,320,079 | 13.03 |
| Simple repeat | 11,884,942 | 1.63 | 0 | 0 | 11,884,942 | 1.63 |
| Unknown | 8,204,034 | 1.12 | 0 | 0 | 8,204,034 | 1.12 |
| Total | 349,596,427 | 47.78 | 69,005,392 | 9.43 | 354,679,850 | 48.47 |
