# Supplementary material for: Whole genome sequencing of a snailfish from the Yap Trench (~7,000 m) clarifies the molecular mechanisms underlying adaptation to the deep sea
Source: PLoS Genet. 2021 May 13;17(5):e1009530. doi: 10.1371/journal.pgen.1009530 (PMC8118300; doi:10.1371/journal.pgen.1009530)
Supplement: S11 Table — (PDF) [file pgen.1009530.s020.pdf]

S11 Table. Statistics of the noncoding RNA in the Yap hadal snailfish genome.

Type		Number	Average length (bp)	Total length (bp)	% of genome
miRNA		1,651	107.67	177,759	0.022429
tRNA		9,143	82.48	754,147	0.10306
rRNA	rRNA	3,572	118.95	424,874	0.058083
	18S	2,707	93.67	253,565	0.034652
	28S	340	347.49	118,145	0.016146
	5.8S	42	150.21	6,309	0.000862
	5S	483	97.01	46,855	0.006403
snRNA	snRNA	1,323	136.87	181,085	0.024747
	CD-box	262	105.79	27,718	0.003788
	HACA-box	152	157.49	23,938	0.003271
	splicing	869	141.69	123,127	0.016826
Total	-	20,544	-	2,137,522	0.292111
