# Supplementary material for: Whole genome sequencing of a snailfish from the Yap Trench (~7,000 m) clarifies the molecular mechanisms underlying adaptation to the deep sea
Source: PLoS Genet. 2021 May 13;17(5):e1009530. doi: 10.1371/journal.pgen.1009530 (PMC8118300; doi:10.1371/journal.pgen.1009530)
Supplement: S12 Table — (PDF) [file pgen.1009530.s021.pdf]

S12 Table. Prediction of the gene structure in the Yap hadal snailfish genome.

Gene set		Number	Average transcript length (bp)	Average CDS length (bp)	Average exons per gene	Average intron length (bp)
De novo	Augustus	36,404	7,416.78	1,182.51	6.50	1,132.99
	GlimmerHMM	121,413	6,046.06	618.52	3.66	2,039.36
	SNAP	70,320	8,325.18	806.70	4.98	1,888.45
	Geneid	61,556	7,947.94	933.00	4.41	2,058.36
	Genscan	40,464	14,225.07	1,452.56	7.80	1,878.54
**Homolog**	*Dre*	28,362	7,570.47	1,402.73	6.64	1,094.15
	Gac	41,014	5,621.77	1,017.79	5.28	1,076.64
	Hsa	21,915	8,614.11	1,381.58	7.16	1,173.46
	Lcr	33,542	7,487.63	1,354.76	6.50	1,116.06
	Mmu	22,232	8,488.07	1,360.13	7.08	1,172.85
	Oni	38,860	6,180.03	1,223.68	5.59	1,079.56
	Tni	25,602	8,143.45	1,339.64	7.22	1,093.05
	Tru	32,858	6,659.68	1,264.91	5.98	1,083.81
**RNAseq**	PASA	96,601	6,658.02	1,045.17	6.70	984.93
	Cufflinks	57,534	12,016.39	2,920.45	8.37	1,233.84
**EVM**		30,938	9,449.83	1,300.12	7.59	1,237.26
**Pasa-update**		30,282	9,858.55	1,348.63	7.82	1,247.13
**Final set**		24,329	10,675.78	1,420.88	8.41	1,249.54

Abbreviation: *Danio rerio* (*Dre*), *Gasterosteus aculeatus* (*Gac*), *Homo sapiens* (*Hsa*), *Ictalurus punctatus* (*Ipu*), *Lepisosteus oculatus* (*Loc*), *Oreochromis niloticus* (*Oni*), *Takifugu rubripes* (*Tru*), *Tetraodon nigroviridis* (*Tni*), *Larimichthys crocea* (*Lcr*), *Mus musculus (Mmu).*
