# Supplementary material for: Whole genome sequencing of a snailfish from the Yap Trench (~7,000 m) clarifies the molecular mechanisms underlying adaptation to the deep sea
Source: PLoS Genet. 2021 May 13;17(5):e1009530. doi: 10.1371/journal.pgen.1009530 (PMC8118300; doi:10.1371/journal.pgen.1009530)
Supplement: S13 Table — (PDF) [file pgen.1009530.s022.pdf]

S13 Table. Information for the RNA-seq data from different Yap hadal snailfish tissues.

ID	Tissues	Raw Reads	Clean reads	Clean bases (Gb)	Error (%)	Q20 (%)	Q30 (%)	GC (%)
A	Liver_1	24,469,958	24,089,587	3.6	0.04	96.0	91.1	48.8
B	Liver_2	24,469,958	24,089,587	3.6	0.05	94.8	88.5	49.0
C	Intestines_1	27,623,319	27,243,693	4.1	0.04	96.4	91.6	51.4
D	Intestines_2	27,623,319	27,243,693	4.1	0.05	95.8	90.3	51.5
E	Eye_1	23,601,154	23,284,416	3.5	0.04	96.4	91.5	50.6
F	Eye_2	23,601,154	23,284,416	3.5	0.05	95.7	90.0	50.7
G	Skin_1	21,010,608	20,692,043	3.1	0.04	96.7	92.0	51.8
H	Skin_2	21,010,608	20,692,043	3.1	0.04	96.1	90.6	51.8
I	Stomach_1	26,402,614	25,951,878	3.9	0.04	96.3	91.4	50.9
J	Staomach_2	26,402,614	25,951,878	3.9	0.05	95.4	89.6	51.0
K	Muscle_1	24,889,824	24,608,914	3.7	0.04	96.7	92.2	52.4
L	Muscle_2	24,889,824	24,608,914	3.7	0.04	96.1	90.7	52.5
