# Supplementary material for: Whole genome sequencing of a snailfish from the Yap Trench (~7,000 m) clarifies the molecular mechanisms underlying adaptation to the deep sea
Source: PLoS Genet. 2021 May 13;17(5):e1009530. doi: 10.1371/journal.pgen.1009530 (PMC8118300; doi:10.1371/journal.pgen.1009530)
Supplement: S14 Table — (PDF) [file pgen.1009530.s023.pdf]

S14 Table. Gene structures of Yap hadal snailfish and other teleost genomes.

Species	Number	Average transcript length (bp)	Average CDS length (bp)	Average exons Per gene	Average exon length (bp)	Average intron length (bp)
Yap hadal snailfish	24,329	10,675.78	1,420.88	8.41	169.02	1,249.54
Mariana hadal snailfish	25,262	10,976.09	1,493.65	9.00	165.94	1,185.17
Tanaka's snailfish	23,776	8,930.18	1,544.21	8.89	173.62	935.64
Zebrafish	25,619	25,207.59	1,642.64	9.42	174.39	2,798.97
Stickleback	20,787	8,451.06	1,548.67	10.40	148.94	734.44
Croaker	22,274	13,224.04	1,740.60	10.48	166.04	1,210.97
Nile tilapia	21,437	14,903.11	1,714.22	10.90	157.25	1,332.07
Pufferfish	19,602	6,066.17	1,516.59	10.52	144.20	478.02
Fugu	18,523	7,492.75	1,693.53	11.10	152.61	574.33
