# Supplementary material for: Whole genome sequencing of a snailfish from the Yap Trench (~7,000 m) clarifies the molecular mechanisms underlying adaptation to the deep sea
Source: PLoS Genet. 2021 May 13;17(5):e1009530. doi: 10.1371/journal.pgen.1009530 (PMC8118300; doi:10.1371/journal.pgen.1009530)
Supplement: S15 Table — (PDF) [file pgen.1009530.s024.pdf]

S15 Table. Functional annotation of the genes in the Yap hadal snailfish genome.

Parameter	Number	Percentage (%)
Total	24,329	-
Swissprot	22,208	91.28
Nr	23,626	97.11
KEGG	20,200	83.03
InterPro	24,196	99.45
GO	22,460	92.32
PFAM	19,164	78.77
Annotated	24,265	99.74
Unannotated	64	0.26
