# Supplementary material for: Whole genome sequencing of a snailfish from the Yap Trench (~7,000 m) clarifies the molecular mechanisms underlying adaptation to the deep sea
Source: PLoS Genet. 2021 May 13;17(5):e1009530. doi: 10.1371/journal.pgen.1009530 (PMC8118300; doi:10.1371/journal.pgen.1009530)
Supplement: S16 Table — (PDF) [file pgen.1009530.s025.pdf]

S16 Table. Accession numbers for the genome assemblies used in this study.

Species	Common name	Accession number
Liparis tanakae	Tanaka's snailfish	GCA_006348945.1
Astyanax mexicanus	Mexican tetra	GCA_000372685.2
Cyprinus carpio	Common carp	GCA_000951615.2
Danio rerio	zebrafish	GCA_000002035.4
Electrophorus electricus	Electric Eel	GCA_013358815.1
Gadus morhua	Atlantic cod	GCA_902167405.1
Gasterosteus aculeatus	Stickleback	GCA_006229165.1
Homo sapiens	Human	GCA_000001405.28
Ictalurus punctatus	Channel catfish	GCA_001660625.1
Lepisosteus oculatus	Spotted gar	GCA_000242695.1
Nothobranchius furzeri	Turquoise killifish	GCA_001465895.2
Oreochromis niloticus	Nile tilapia	GCA_001858045.3
Oryzias melastigma	Marine medaka	GCA_002922805.2
Salmo salar	Atlantic Salmon	GCA_000233375.4
Takifugu rubripes	Fugu	GCA_901000725.2
Tetraodon nigroviridis	Spotted pufferfish	GCA_000180735.1
Xiphophorus maculatus	Platyfish	GCA_002775205.2
Larimichthys crocea	Large yellow croaker	GCA_000972845.2
Stegastes partitus	Bicolor damselfish	GCA_000690725.1
Pseudoliparis swirei	Mariana hadal snailfish	PRJNA472845
