# Supplementary material for: Whole genome sequencing of a snailfish from the Yap Trench (~7,000 m) clarifies the molecular mechanisms underlying adaptation to the deep sea
Source: PLoS Genet. 2021 May 13;17(5):e1009530. doi: 10.1371/journal.pgen.1009530 (PMC8118300; doi:10.1371/journal.pgen.1009530)
Supplement: S17 Table — (PDF) [file pgen.1009530.s026.pdf]

S17 Table. Partial GO enrichment (Level 4) of positively selected genes from the genome assembly of Yap hadal snailfish.

GO ID	GO Term	GO Class	*P*-value	Adjusted *P*-value	Gene number
GO:0016741	transferase activity, transferring one-carbon groups	MF	0.0001376	0.2409646	25
GO:0044428	nuclear part	CC	0.002388	0.4312696	29
GO:0003676	nucleic acid binding	MF	0.00267	0.4312696	304
GO:0006281	DNA repair	BP	0.0031379	0.4312696	34
GO:0016810	hydrolase activity, acting on carbon-nitrogen (but not peptide) bonds	MF	0.003553	0.4312696	14
GO:0043244	regulation of protein complex disassembly	BP	0.0049101	0.4344086	3
GO:0070013	intracellular organelle lumen	CC	0.0055698	0.4344086	27
GO:0044391	ribosomal subunit	CC	0.0072305	0.4661267	6
GO:0008023	transcription elongation factor complex	CC	0.012441	0.4964212	3
GO:0008406	gonad development	BP	0.012447	0.4964212	2
GO:0034641	cellular nitrogen compound metabolic process	BP	0.0231437	0.4964212	356
GO:0008219	cell death	BP	0.0233498	0.4964212	21
GO:0008073	ornithine decarboxylase inhibitor activity	MF	0.0238081	0.4964212	2
GO:1901360	organic cyclic compound metabolic process	BP	0.0309903	0.4964212	352
GO:0009888	tissue development	BP	0.031599	0.4964212	3
GO:0006725	cellular aromatic compound metabolic process	BP	0.0325971	0.4964212	348
GO:0046483	heterocycle metabolic process	BP	0.032734	0.4964212	349
GO:0016645	oxidoreductase activity, acting on the CH-NH group of donors	MF	0.039052	0.4964212	5
GO:0016627	oxidoreductase activity, acting on the CH-CH group of donors	MF	0.0444156	0.4964212	7
