# Supplementary material for: Whole genome sequencing of a snailfish from the Yap Trench (~7,000 m) clarifies the molecular mechanisms underlying adaptation to the deep sea
Source: PLoS Genet. 2021 May 13;17(5):e1009530. doi: 10.1371/journal.pgen.1009530 (PMC8118300; doi:10.1371/journal.pgen.1009530)
Supplement: S18 Table — (PDF) [file pgen.1009530.s027.pdf]

S18 Table. The positively selected genes involved in the DNA repair from Yap hadal snailfish.

	Gene ID	Name	Description
1	Contig538_pilon.10	*ssna1*	Sjogren syndrome nuclear autoantigen 1
2	Contig31_pilon.44.1	*blm*	bloom syndrome protein
3	Contig31_pilon.277	*tobi*	target of brain insulin
4	Contig31_pilon.176	*rad52*	DNA repair protein RAD52 homolog
5	Contig150_pilon.2	*tymp*	thymidine phosphorylase
6	Contig859_pilon.172	*nphs1*	Nephrin
7	Contig210_pilon.12	*hes7*	hairy and enhancer of split 7
8	Contig1453_pilon.52	*ino80e*	INO80 complex subunit E
9	Contig17_pilon.57	*rtn3*	Reticulon-3
10	Contig173_pilon.124.1	*ttll11*	tubulin tyrosine ligase-like family member 11
11	Contig2614_pilon.17.1	*rad9a*	cell cycle checkpoint control protein RAD9A
12	Contig1218_pilon.3	*orc4*	origin recognition complex subunit 4
13	Contig405_pilon.32	*nhej1*	non-homologous end-joining factor 1
14	Contig1236_pilon.75	*ercc1*	DNA excision repair protein ERCC-1
15	Contig2450_pilon.51	*gramd1c*	-GRAM domain-containing protein 1C
16	Contig1232_pilon.3	*mbd4*	methyl-CpG-binding domain protein 4
17	Contig79_pilon.12	*pms1*	DNA mismatch repair protein PMS1
18	Contig132_pilon.13	*rexo1*	RNA exonuclease 1 homolog
19	Contig77_pilon.52	*tasp1*	Threonine aspartase 1
20	Contig77_pilon.31.17	*nsmce4a*	Non-structural maintenance of chromosomes element 4 homolog A
21	Contig1371_pilon.1	*fancl*	E3 ubiquitin-protein ligase FANCL
22	Contig446_pilon.62	*gtf2h3*	General transcription factor IIH subunit 3
23	Contig423_pilon.55	*exo1*	exonuclease 1
24	Contig1717_pilon.109	*phr*	deoxyribodipyrimidine photo-lyase
25	Contig984_pilon.15	*sprtn*	SprT-like domain-containing protein Spartan
26	Contig1085_pilon.20.1	*dmap1*	DNA methyltransferase 1-associated protein 1
27	Contig622_pilon.196	*aste1*	Protein asteroid homolog 1
28	Contig622_pilon.209	*twistnb*	DNA-directed RNA polymerase I subunit RPA43
29	Contig1049_pilon.1	*ascl1a*	achaete-scute complex protein
30	Contig517_pilon.60	*mgmt*	Methylated DNA protein cysteine methyltransferase
31	Contig80_pilon.23	*polk*	DNA polymerase kappa
32	Contig966_pilon.20	*fam73a*	Protein FAM73A
33	Contig2719_pilon.14	*cpb2*	carboxypeptidase B2
34	Contig1619_pilon.27	*nom1*	nucleolar MIF4G domain-containing protein 1
