# Supplementary material for: Whole genome sequencing of a snailfish from the Yap Trench (~7,000 m) clarifies the molecular mechanisms underlying adaptation to the deep sea
Source: PLoS Genet. 2021 May 13;17(5):e1009530. doi: 10.1371/journal.pgen.1009530 (PMC8118300; doi:10.1371/journal.pgen.1009530)
Supplement: S19 Table — (PDF) [file pgen.1009530.s028.pdf]

S19 Table. List of Pfam domains with more copies in Yap hadal snailfish than in other species.

Domain name	Yap hadal snailfish	Mariana hadal snailfish	Tanaka's snailfish	Zebrafish
Ion_trans	182	172	164	154
Pro_isomerase	27	22	19	19
Dynein_heavy	20	12	15	13
ASC	18	10	10	7
Nebulin	11	6	7	4
Fam20C	10	9	4	6
Sarcoglycan_1	9	7	5	4
DUF3498	9	3	3	3
TMPIT	9	2	3	3
Beta-Casp	8	6	4	4
Peptidase_S10	8	3	4	4
Rad51	8	4	6	7
DUF4487	7	10	1	1
Nuc_rec_co-act	7	5	4	3
BPS	7	4	4	3
SRC-1	7	4	3	3
Orn_DAP_Arg_deC	7	7	4	3
zf-nanos	7	3	3	3
RNA_pol_Rpb4	7	4	1	2
FG-GAP_2	6	5	4	2
Ribosomal_L3	6	5	3	2
Rad21_Rec8	6	5	3	3
RMMBL	6	5	3	3
DUF3399	6	3	3	3
CRF	6	3	3	3
ALMS_motif	5	4	3	1
NUC194	5	2	1	1
EnY2	4	4	0	1
Keratin_B2_2	3	0	2	0
B_lectin	3	1	1	0
TMEM100	3	2	2	0
