# Supplementary material for: Whole genome sequencing of a snailfish from the Yap Trench (~7,000 m) clarifies the molecular mechanisms underlying adaptation to the deep sea
Source: PLoS Genet. 2021 May 13;17(5):e1009530. doi: 10.1371/journal.pgen.1009530 (PMC8118300; doi:10.1371/journal.pgen.1009530)
Supplement: S20 Table — (PDF) [file pgen.1009530.s029.pdf]

S20 Table. Numbers of olfactory receptor (OR) genes in the examined fish species.

Species	Group	α	β	γ	γ	ε	ξ	η	θ	κ	T
Yap hadal snailfish	F[a]	0	0	0	14	1	7	2	1	0	25
	T[b]	0	1	0	24	1	7	5	1	1	40
Mariana hadal snailfish	F	0	0	0	14	1	7	2	1	0	25
	T	0	1	0	24	1	7	5	1	1	40
Zebrafish	F	0	1	1	44	11	27	22	1	1	108
	T	0	1	1	53	14	41	24	1	1	136
Pufferfish	F	0	1	0	28	2	6	5	1	1	44
	T	0	1	0	61	2	6	24	1	1	98
Fugu	F	0	1	0	42	2	5	10	1	1	62
	T	0	1	0	62	2	6	22	1	1	95
Stickleback	F	0	1	2	20	2	3	0	0	1	29
	T	0	1	2	26	2	13	0	0	1	45
Croaker	F	0	0	0	52	2	6	12	1	0	73
	T	0	1	0	74	3	9	38	1	1	127
Sia	F	0	1	1	26	4	5	11	2	2	52
	T	0	1	2	61	10	42	21	2	2	141
Sig	F	0	1	1	33	3	11	10	2	2	62
	T	0	1	1	50	8	21	17	2	2	102
Sir	F	0	1	2	27	9	7	11	2	1	60
	T	0	1	2	61	12	53	27	2	2	160

[a] 'F' represents the number of functional OR genes. [b]'T' represents total number of OR genes.

Sia: *Sinocyclocheilus anshuiensis*, Sig: *Sinocyclocheilus grahami*, Sir: *Sinocyclocheilus rhinocerous*.
