# Supplementary material for: Whole genome sequencing of a snailfish from the Yap Trench (~7,000 m) clarifies the molecular mechanisms underlying adaptation to the deep sea
Source: PLoS Genet. 2021 May 13;17(5):e1009530. doi: 10.1371/journal.pgen.1009530 (PMC8118300; doi:10.1371/journal.pgen.1009530)
Supplement: S9 Fig — A frameshift mutation was identified in the YHS genome that led to a severe mutation in the open reading frame (ORF) of the ANNAT gene. The translated protein according to the mutated ORF is completely distinct from that of Medaka ANNAT. (PDF) [file pgen.1009530.s009.pdf]

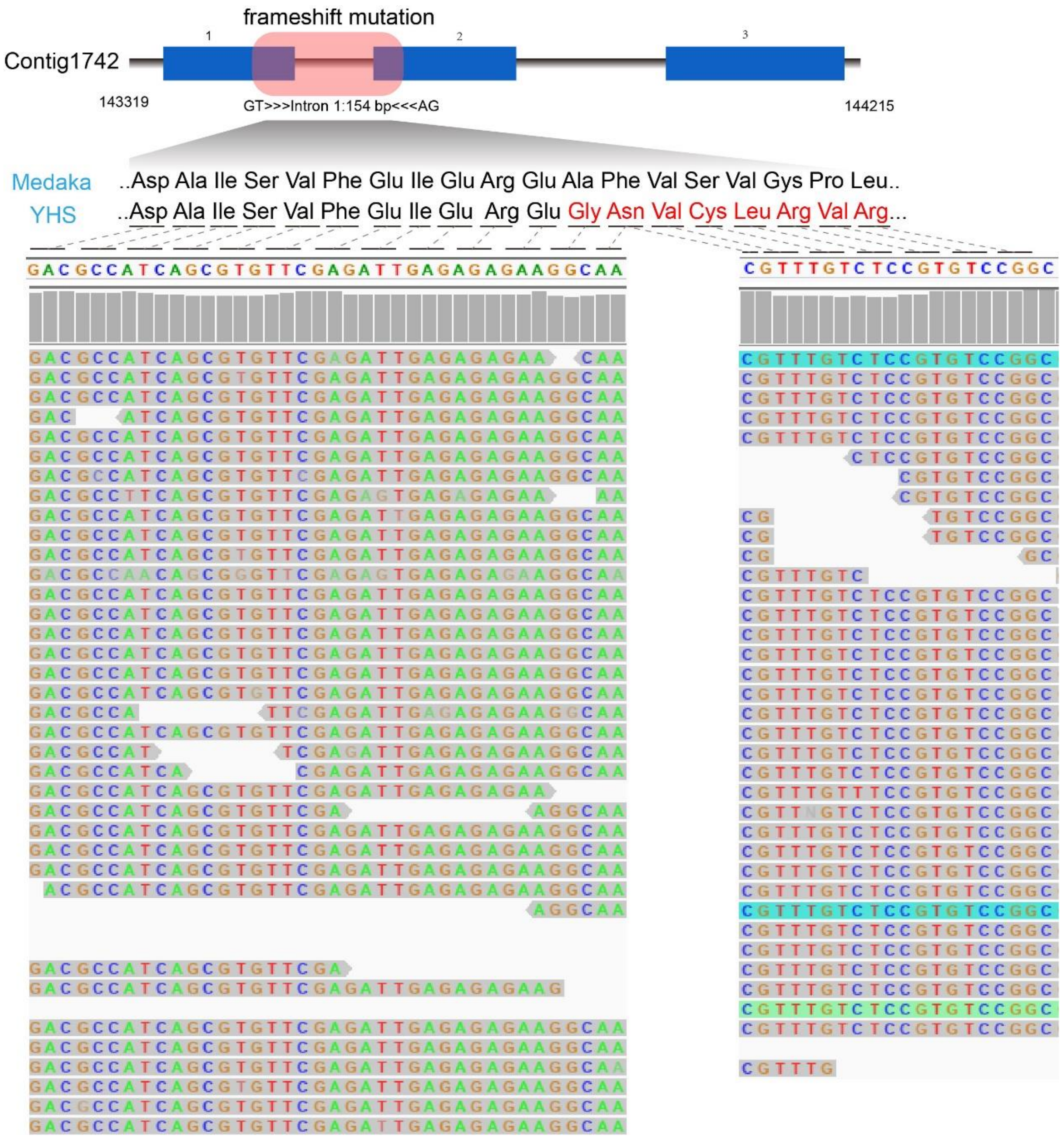

S9 Fig. Frameshift mutation in the ANNAT gene of Yap hadal snailfish (YHS). A frameshift mutation was identified in the YHS genome that led to a severe mutation in the open reading frame (ORF) of the ANNAT gene. The translated protein according to the mutated ORF is completely distinct from that of Medaka ANNAT.
