# Supplementary material for: Whole genome sequencing of a snailfish from the Yap Trench (~7,000 m) clarifies the molecular mechanisms underlying adaptation to the deep sea
Source: PLoS Genet. 2021 May 13;17(5):e1009530. doi: 10.1371/journal.pgen.1009530 (PMC8118300; doi:10.1371/journal.pgen.1009530)
Supplement: S21 Table — (PDF) [file pgen.1009530.s030.pdf]

S21 Table. The copy number of crystalline genes in Yap hadal snailfish and other fish species.

Gene name	Zebrafish	YHS	Fugu	stickleback	croaker	pufferfish	Sig	Sir	Sia
cryaa	1	0	1	1	1	2	3	2	5
cryaba	1	1	1	1	1	1	1	2	2
cryabb	2	1	0	2	3	1	2	2	2
cryba1a	1	6	1	2	2	2	2	2	2
cryba1b	1	0	1	0	0	0	2	2	2
cryba1l1	1	1	1	2	3	2	2	2	1
cryba1l2	1	0	0	0	0	0	4	3	3
cryba2a	1	0	0	0	1	0	2	2	2
cryba2b	1	0	1	1	0	1	3	2	2
cryba4	1	1	1	0	1	1	2	1	2
crybb1	1	1	1	0	1	1	2	2	2
crybb1l1	1	1	2	2	3	2	2	2	2
crybb1l2	1	0	0	0	0	0	2	3	2
crybb1l3	1	6	1	1	1	1	1	1	1
crybb2	1	0	1	1	1	1	2	2	2
crybb3	1	1	2	2	3	2	2	2	2
crybgx	1	1	1	1	1	1	3	3	5
crygm1	0	0	0	0	0	2	2	3	2
crygm2	15	6	2	9	11	11	17	10	9
crygm3	1	0	5	1	2	5	2	2	1
crygm4	1	0	0	0	0	0	0	0	0
crygm5	0	0	0	1	0	0	1	1	1
crygm6	1	0	1	0	1	1	2	1	1
crygm7	1	0	0	1	1	0	1	1	0
crygmx	1	1	1	1	1	1	1	1	0
crygmxl1	1	0	1	1	1	1	1	1	1
crygmxl2	1	0	0	0	1	0	2	2	2
crygn1	1	2	1	1	2	1	2	2	2
crygn2	1	0	1	1	1	1	1	2	1
crygs1	1	1	0	1	1	1	5	2	3
crygs2	1	0	0	0	0	0	2	2	2
crygs3	1	0	4	1	3	3	3	3	4
crygs4	1	0	0	1	0	0	1	2	2
cryl1	1	1	1	1	1	0	2	2	2
crym	1	1	1	1	1	1	2	2	1
cryz	1	2	0	1	1	1	1	1	1
cryzl1	1	1	1	1	1	0	2	2	2
total	50	**35**	34	39	51	47	87	77	76

Sia: *Sinocyclocheilus anshuiensis*, Sig: *Sinocyclocheilus grahami*, Sir: *Sinocyclocheilus rhinocerous*, YHS: Yap hadal snailfish.
